# Intra-tumoral spatial heterogeneity in breast cancer quantified using high-dimensional protein multiplexing and single cell phenotyping

**DOI:** 10.1186/s13058-025-02038-1

**Published:** 2025-05-21

**Authors:** Alison M. Cheung, Dan Wang, Mary Anne Quintayo, Yulia Yerofeyeva, Melanie Spears, John M. S. Bartlett, Lincoln Stein, Jane Bayani, Martin J. Yaffe

**Affiliations:** 1 Biomarker Imaging Research Lab (BIRL), Sunnybrook Research Institute, Rm S658, 2075 Bayview Avenue, Toronto, ON Canada; 2 Diagnostic Development, Ontario Institute for Cancer Research, Toronto, ON Canada; 3 Department of Laboratory Medicine and Pathobiology, University of Toronto, Toronto, ON Canada; 4 University of Edinburgh, Edinburgh, UK; 5 Informatics and Bio-Computing, Ontario Institute for Cancer Research, Toronto, ON Canada; 6 Department of Medical Biophysics, University of Toronto, Toronto, ON Canada

**Keywords:** Cancer heterogeneity, Intra-tumoral heterogeneity, High-dimensional protein multiplexing, Single cell phenotyping, Spatial heterogeneity, Cancer biomarker

## Abstract

**Background:**

Breast cancer is a highly heterogeneous disease where variations of biomarker expression may exist between individual foci of a cancer (intra-tumoral heterogeneity). The extent of variation of biomarker expression in the cancer cells, distribution of cell types in the local tumor microenvironment and their spatial arrangement could impact on diagnosis, treatment planning and subsequent response to treatment.

**Methods:**

Using quantitative multiplex immunofluorescence (MxIF) imaging, we assessed the level of variations in biomarker expression levels among individual cells, density of cell cluster groups and spatial arrangement of immune subsets from regions sampled from 38 multi-focal breast cancers that were processed using whole-mount histopathology techniques. Molecular profiling was conducted to determine the intrinsic molecular subtype of each analysed region.

**Results:**

A subset of cancers (34.2%) showed intra-tumoral regions with more than one molecular subtype classification. High levels of intra-tumoral variations in biomarker expression levels were observed in the majority of cancers studied, particularly in Luminal A cancers. HER2 expression quantified with MxIF did not correlate well with HER2 gene expression, nor with clinical HER2 scores. Unsupervised clustering revealed the presence of various cell clusters with unique IHC4 protein co-expression patterns and the composition of these clusters were mostly similar among intra-tumoral regions. MxIF with immune markers and image patch analysis classified immune niche phenotypes and the prevalence of each phenotype in breast cancer subtypes was illustrated.

**Conclusions:**

Our work illustrates the extent of spatial heterogeneity in biomarker expression and immune phenotypes, and highlights the importance of a comprehensive spatial assessment of the disease for prognosis and treatment planning.

**Supplementary Information:**

The online version contains supplementary material available at 10.1186/s13058-025-02038-1.

## Background

Heterogeneity is a noted feature in cancer and has a potential impact on cancer diagnosis, decision-making for therapy and resistance to treatment [1–4]. While morphological differences in the cancer epithelium is a recognized pathologic feature, biomarker presence and intensity are generally measured in the clinical pathology lab by a bulk assessment derived from immunohistochemical (IHC) staining and described by a single (histo) score. This score combines the percentage of positive cancer cells and the average staining intensity for the entire cancer lesion being studied. Spatial heterogeneity of markers is rarely quantified.

Intra-tumoral heterogeneity, as measured based on the diversity in the genetic landscape of cancer cells, has been studied intensively in the research lab setting [5–12]. Clusters of cells with various genetic “markers” have been identified, highlighting the marked level of non-homogeneous composition of cells in the cancer lesion. Recently, the use of single-cell phenotyping with protein multiplexing and single-cell transcriptomics, together with methods for spatial biology, have facilitated efforts for better characterizing these subgroups of cells. Nevertheless, the degree of variations among invasive foci in a multi-focal cancer, or between different regions extracted from the same uni-focal cancer lesion is not normally quantified. Histopathology of surgical specimens in the anatomic pathology lab is usually limited to processing and assessing one or two tumor slices representing the largest detected cancer lesion. Thus, the characteristics of other small or occult lesions in the same specimen are not usually studied.

Molecular assays have also revealed an additional level of complexity of breast cancer heterogeneity. Molecular intrinsic subtypes of breast cancer with PAM50 classification identified four major molecular subtypes: Luminal A, Luminal B, HER2-enriched and Basal-like [13]. This classification has assisted in stratifying hormonal receptor (HR)-positive cancers into Luminal A and Luminal B subgroups, with Luminal A cancers expressing both ER and PR, while Luminal B cancers are usually higher grades with expression of ER and with Ki67 positivity higher than 14% [14, 15]. While HR-positive cancers are better defined with molecular assays, basal-like cancers are also noted for their molecular heterogeneity. A majority of the ER, PR and HER2-negative (triple-negative breast cancers, TNBC) are molecularly subtyped as basal-like, but it is reported that there are multiple “TNBCtype”s [16], suggesting that a spectrum of subgroups exists within TNBC [17–19].

Platforms that allow multiplexed staining of protein markers and quantitative image analysis of their spatially dependent expression are now available. Here, we sought to take an extensive examination of heterogeneity using immunofluorescence protein multiplexing (MxIF) to investigate the spatial heterogeneity of protein expression as well as to characterize the localization of immune subsets in the lesion. The analyses were performed on whole mount (WM) sections produced from lumpectomies for breast cancer [20]. WM processed lumpectomies allow for multiple regions from the entire lesion to be studied, building an excellent collection of tissue specimens for the assessment of intra-tumoral heterogeneity and comparison between breast cancer subtypes.

## Methods and materials

### Lumpectomy specimens and tissue microarray

Sixty-one WM surgical specimens of lumpectomy from the *Genomic-Imaging Neo-pathologic Analysis* cohort (*GINA) of* women diagnosed with breast cancer at the Sunnybrook Health Sciences Centre (Toronto, ON, Canada) from 2006 to 2014 were included in the construction of tissue microarrays (TMA) in this study. All specimens were processed by the Biomarker Imaging Research Lab (BIRL) at the Sunnybrook Research Institute (Toronto, ON) (ethics REB#192–2009) and TMA construction, nucleic acid extraction and molecular profiling were performed at the Ontario Institute for Cancer Research (Toronto, ON) (REB#36112). Molecular and MxIF studies of this cohort received research ethics approval from the Research Ethics Board at Sunnybrook Health Sciences Centre (REB#292–2014). The complete surgical specimen was processed, sliced and micro-sectioned to produce 4 μm sections. These were mounted on large (up to 12.5 cm x 17.5 cm) glass slides. Each tissue section was stained with Haematoxylin and Eosin (H&E) followed by digitization at 0.5 μm per pixel, allowing the pathologist to assess all tissues in the lumpectomies without the need for tiling [20].

Cancer lesions from each WM tissue section were annotated by research pathologist (K.L.) [21]. The definition of multi-focality in our cohort is given in Supplemental Information (Methods). Tissue microarrays for all of the cancer foci were prepared after careful selection of regions for ER and PR staining intensity as well as cancer cellularity. For each lumpectomy, multiple cores were collected from various regions of each focus when feasible and from each focus of multi-focal cancers. The number of regions per lumpectomy that were included in the study ranged from 2 to 9 (Table 1). The original cohort included whole-mount lumpectomies from 61 women and these were all used in the construction of 3 tissue microarray blocks which were all studied with protein multiplexing and molecular subtyping. However, inspection subsequent to multiplexing revealed that due to damage or insufficient number of cells in the cores only 38 cases were suitable for comparison to assess heterogeneity between distinct regions. Figure S.1 in Supplemental information illustrates the study workflow.

**Table 1:**
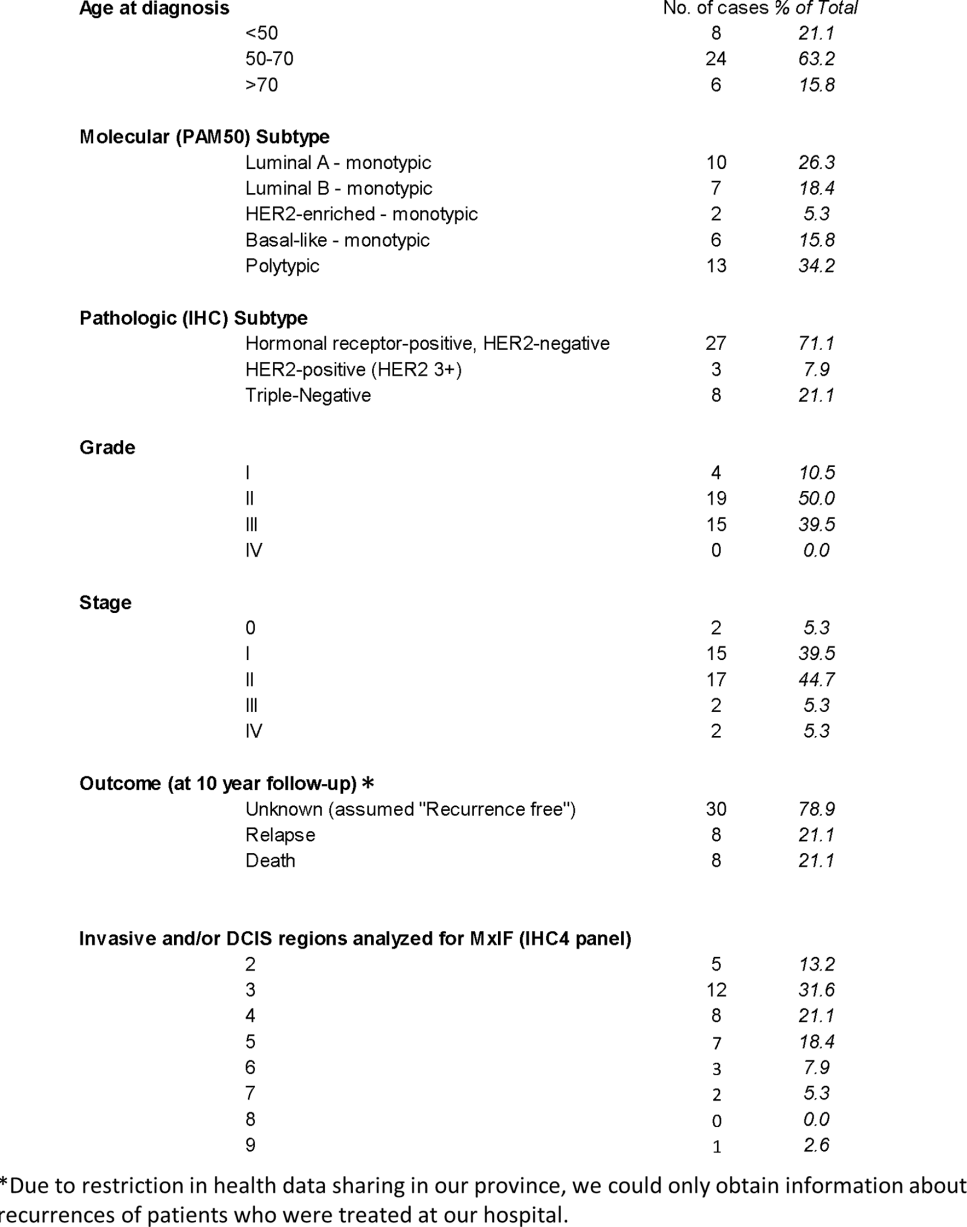
Clinical information of lumpectomy cases included in the study

### Antibody selection, conjugation, calibration and validation

Commercially available antibodies for each protein marker of interest were first tested using standard IHC with appropriate positive and negative control tissues. Antibodies that showed specific staining and intensity comparable to IHC were selected and conjugated to Cy3 or Cy5 fluorophores following established protocols [22]. Each fluorophore-conjugated antibody was optimized and validated using control tissues. The staining patterns, specificities and intensities of conjugated antibodies were compared to the optimized IHC staining of the same tissue and evaluated by study pathologists. Detailed steps in MxIF staining are described in Supplemental Information (Methods). For breast biomarkers of ER, PR, HER2 and Ki67, we used clinically annotated breast cancer tissues for antibody validation. The specific clones of breast biomarkers used in this study were: ER (*SP1*,* Spring Biosciences*), PR (*PR1294*,* Dako*), HER2/neu (*4B5*,* Roche)* and Ki67 (*SP6*, Zeta Corporation). A range of concentrations was tested for each fluorophore-conjugated antibody (or unconjugated antibodies in the case of PR and HER2) in the MxIF staining protocol and the parameters that resulted in staining intensities similar to IHC performed in the clinical immunohistochemistry lab were selected [23] (Supplemental information table S.I). For the Immune panel, the following protein markers were studied: CD3 (*F7.2.38*,* Dako*), CD8 (*C8/144B*,* Dako*), CD68 (*KP-1*,* ThermoFisher*), CD163 (*EDHU-1*,* BioRad*), FoxP3 (*150D*,* BioLegend*), PD1 (*EPR4877 *(2), *Abcam*), PDL1 (*SP263*,* Roche*) (Table S.I). Tonsil tissue was added to the TMA tissue sections for protein multiplex studies of the Immune panel to serve as positive on-slide tissue controls.

### Immunofluorescence protein multiplexing (MxIF)

Protein multiplexing was conducted on the MxIF (GE Research (GER), Niskayuna, NY, USA) for the IHC4 panel (ER, PR, HER2, Ki67 and CK8/18), and on the commercial platform (Cell DIVE, Leica Biosystems), a successor product to the GER technology, for the immune panel (CD3, CD8, CD68, CD163, PD-1, PDL-1, Ki67, FoxP3, CK8/18). For both systems, fluorophore-conjugated antibodies were sequentially applied onto a single tissue section of the TMA (a pair of Cy3- and Cy5-conjugated antibodies on each staining round), followed by image acquisition and chemical bleaching to inactivate optical signals from the antibodies (Supplemental information: Methods). The order of antibody staining for IHC4 and immune panels is listed in the Supplemental Information (Table S.I). In the first round of staining, unconjugated antibodies for HER2 and PR (IHC4 panel), PDL-1 (Immune panel) were applied in primary incubation, followed by secondary staining with fluorophore conjugates of Cy3 or Cy5 respectively. As these antibodies were only available in diluted, ready-to-use format custom in-house fluorophore conjugation was not feasible. All other antibodies were applied as fluorophore-conjugated antibodies. The pixel size for MxIF at 20x image acquisition is 0.293 μm. The pixel size for Cell DIVE at 20x image acquisition is 0.325 μm with intensities presented on a 16-bit linear scale.

### Image analysis, single cell segmentation and data post-processing

Following each immunofluorescence multiplexing experiment, the quality of staining of each TMA core was visually inspected with GE Layers (image analysis software for MxIF developed by GE Research) for the IHC4 panel or with HALO (digital pathology imaging software, Indica Labs) for the Immune panel. Cores that showed damaged tissue were excluded from quantitative analysis. Image registration and single cell segmentation were performed using the Single Cell Metrics plugin (developed by GE Research) in image analysis software FIJI [24] for the IHC4 panel. The Single Cell Metrics plugin uses signals of DAPI for nuclear segmentation based on blobness detection. Parameters in the plugin allowed for adjusting the size of the cell boundary to capture finer or larger structures [25]. Single cell segmentation for the Immune panel was conducted with HALO. For both platforms, single cell measurements of cell size, protein marker expression level (raw pixel intensity) and x, y coordinates were collected. Cells with DAPI signal intensities of less than 1000 were excluded to limit analysis to undamaged cells. Log_2_ transformation and normalization (on a scale of 0–15 for IHC4, Z-score for Immune markers) were applied to the raw signal intensity for each marker.

### Histoscore (H-score), HER2 scoring and Ki67-positivity

Expression of ER and PR in each cell was categorized to “1+” (> 0 to  < 7 of range normalised (0–15) expression), “2+” (7 to 12) and “3+” (> 12 to max) (Supplemental information Fig. (S.2). For HER2, cells were categorized to “0” if their range normalised expression level are from 0 to 5, “1+” for levels from > 5 to 10, “2+” for levels from > 10 to 13 and “3+” for levels from 13-max (Fig. S.2). Threshold levels were determined after consideration of the overall distribution of the spectrum of intensity levels in cancer cells of hormonal receptor-positive cases and comparison to cases that were clinically scored. After levels of 1+, 2 + and 3 + were determined for each protein, Histoscores (H-score) for the TMA core were then calculated using the formula H = 3 x (% of cells with 3 + staining) + 2 x (% of cells with 2 + staining) + (% of cells with 1 + staining) [26]. HER2 clinical IHC scores were extracted from pathology reports of GINA study participants at the Sunnybrook Health Sciences Centre [20]. HER2 IHC and in situ hybridization (ISH) were performed with clinically validated standard procedures (Supplemental Information: Methods). For Ki67, any cancer cell with normalized expression level of Ki67 > 0 was considered ti be positive, to align with clinical IHC Ki67 scoring protocol where any amount of brown nuclear staining in a cancer cell is considered positive [27].

### Thresholding and classification of immune lineage cells

Binary classification (positive vs. negative) of immune cell type was conducted by thresholding of protein marker signals [28]. A threshold for each protein marker was determined by comparing the signals from single cells across the TMA with the tonsil control tissue.

### Statistical analysis

Unsupervised clustering was performed using PhenoGraph [29], a K-nearest neighbor and graph approach in assigning cells to various clusters based on their protein marker co-expression signature. The iCellR package in R was used, with K = 300 applied for IHC4 co-expression clusters and K = 30 applied for Immune spatial clusters. The optimal number of clusters is determined by testing different values of K and maximizing the value of modularity provided by PhenoGraph for each clustering output. The value of modularity ranges from − 1 to 1 and the higher value the better quality of the graphs/cluster assignments (Supplemental Information: Methods). A dendrogram was built in R using the package “heatmaply”. Dimensional-reduction visualizations using t-distributed stochastic neighbor embedding (t-SNE) was conducted using R Statistics (package “Rtsne”). Levels of significant difference in ER and PR Histoscores and % of Ki67 + cells comparison among molecular subtypes of cancers, and in immune spatial features comparison among molecular subtype cancers and cores, were determined using ANOVA tests (95% confidence level) in R statistics with aov (package “stats, version 3.6.2). Level of correlation between HER2 RNA expression and HER2 MxIF-measured Histoscore was conducted using Pearson and Spearman correlation in R statistics. Comparison of the immune phenotype (immune spatial features patch composition) between Lum A cores and Lum B cores in polytypic cancers was conducted with 2-sample Wilcoxon rank sum test wilcox.test (R package “stats”) at 95% confidence level. Heterogeneity was quantified using Rao’s quadratic entropy (raoD) in R Statistics (package “picante” version 1.8.2). Quadratic Entropy is a measure of how diverse a community is based on the abundance of species and the dissimilarity between species [30]. In our study there is no phylogenetic tree (or dissimilarity distance) applied, therefore the entropy output is equivalent to Simpson’s diversity index [31]. With raoD, “alpha diversity” outputs the average within-community diversity, or a measure of intra-core heterogeneity. “Beta diversity” outputs the average among-community diversity, or a measure of intra-tumoral heterogeneity.

### Molecular analysis

During collection of tissue cores for tissue microarray construction, adjacent cores were taken from the same region for nucleic acid extraction [32]. 300ng of total RNA was used for the NanoString BC360 Assay (NanoString Technologies Inc., Seattle, WA, USA) and processed according to the manufacturers’ directions using the nCounter System. Among these signatures is the PAM50 Signature [33, 34], which classifies tumors into one of four molecular subtypes (Luminal A, Luminal B, HER2-enriched, and Basal-like) [35].

### Spatial analysis using image patches

For the Immune panel, the image of each TMA core was divided into small patches of 150 pixel x 150 pixel (48.75 μm x 48.75 μm). The numbers of each immune subset and cancer cell localized to each patch were counted. The fraction of cancer epithelial (CK8/18+) and 9 immune lineage cells (CD3+/CD8-, CD3+/CD8+, CD68+, CD163+, CD68+/CD163+, CD68+/PDL1+, CD163+/PDL1+, CD3+/CD8+/PD1+, CD3+/CD8-/PD1+) in each patch was used for PhenoGraph clustering to identify immune phenotypes. With PhenoGraph clustering, 12 clusters were generated.

## Results

### A subset of multi-focal breast cancers exhibited more than one molecular subtype

A total of 38 lumpectomy cases, each with more than 1 focus of invasive cancer or DCIS was studied. Table 1 provides a summary of the clinical descriptions of this cohort, including age at diagnosis, cancer grade and stage. For each studied region, 4 tissue cores in close proximity were extracted. Two of the cores were used in TMA construction, one for DNA extraction and the final one for RNA extraction and molecular subtyping (see Materials & Methods). The majority of the cases exhibited consistent, uniform molecular subtype classification (referred to here as “molecularly monotypic”) for all of the regions that were successfully analysed. Accordingly, there were 10 Luminal A cancers, 7 Luminal B, 2 HER2-enriched and 6 Basal-like cancers that are molecularly-monotypic (Table 1). Thirteen of the 38 cancers (34.2%) had regions that were classified as having different molecular subtypes within the cancer. Hereafter in this report these cases will be referred to as molecular-polytypic cancers. Based on receptor IHC classifications, the majority of all cancers were hormonal receptor-positive, HER2-negative cancers (27 of 38, or 71%) and almost two-thirds of the cancers (23 of 38, or 61%) were of grade I or II (Table 1). Of the molecular-polytypic cancers, one case was a clinical IHC-classified HER2 3+ (HR-negative) and two were clinical IHC-classified triple-negative breast cancers (TNBC) (See Table S.II for a detailed description of the IHC-classification and molecular-subtypes classification of the cores and cases). Representative MxIF images of tissue cores of different molecular subtype are shown in Fig. S.3–7 in the Supplemental Information.

### Intra-tumoral heterogeneity of IHC4 marker expressions was observed among regions extracted from the same cancer

**Fig. 1:**
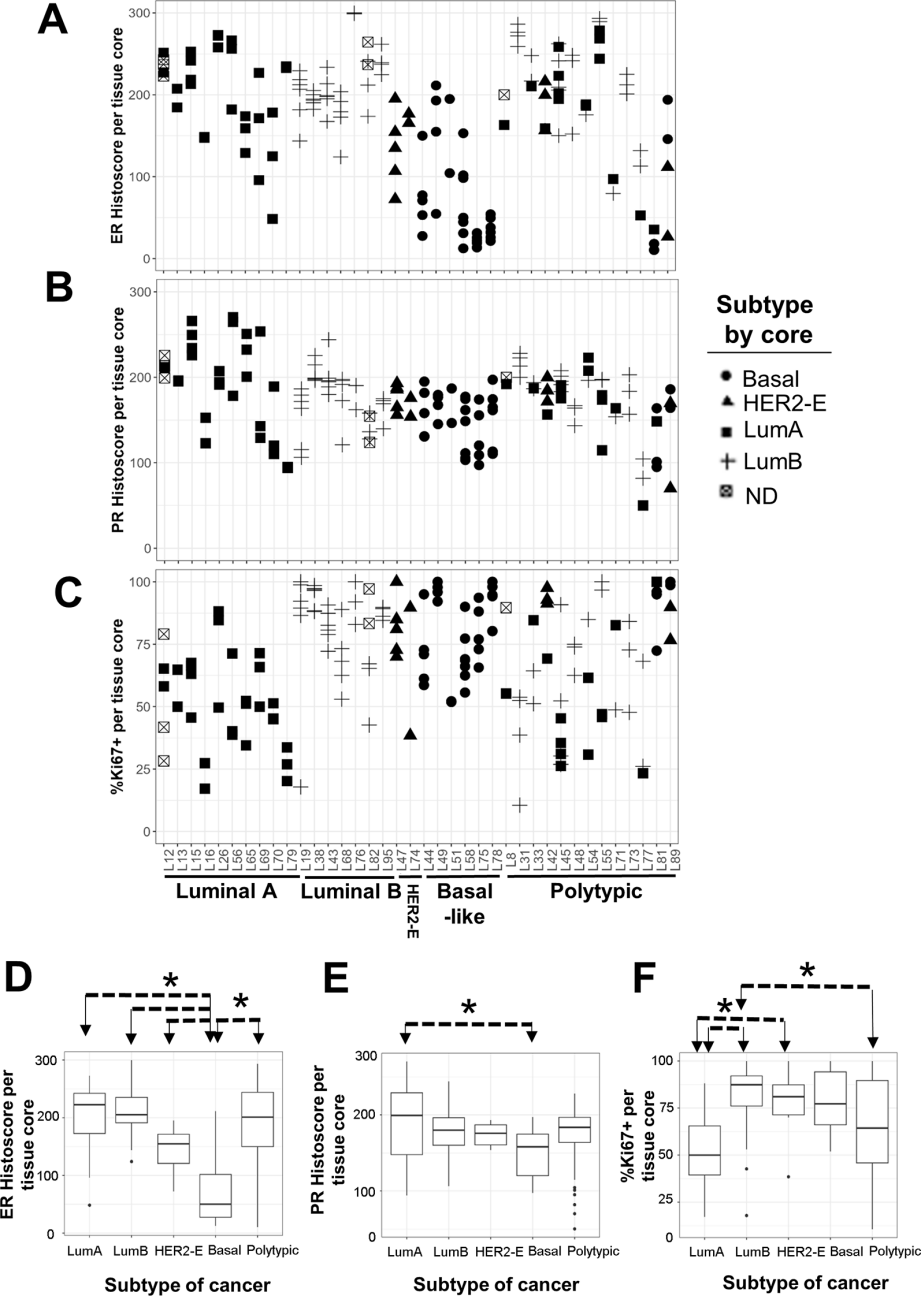
Intra-tumoral heterogeneity of ER, PR and Ki67 in WM-processed breast cancer. (**A**) ER histoscore quantified from MxIF for each cored region per lumpectomy (L) case was shown. Cancers are segregated into molecular subtype and the molecular subtype of the tissue core is indicated by symbols. Cases with more than one molecular subtype were identified as Polytypic. (**B**) PR histoscore quantified from MxIF for each cored region per L case. (**C**) Percentage of MxIF-measured Ki67-positive cells in each cored region per L case. Of the 17 cancers that were classified as molecular-polytypic based on RNA expression, due to tissue damage during MxIF the quality of 4 of these samples was inadequate for protein evaluation of all of the tissue cores and for this reason they were shown in the figure as if they belonged to only one subtype: (L8 – LumA only, L31 – LumB only, L48 – LumB only and L73 – Lum B only). (**D**) Boxplot of ER histoscores from tissue cores in cancers stratified by molecular subtype. (**E**) Boxplot of PR histoscore from tissue cores in cancers stratified by molecular subtype. (**F**) Boxplot of the percentages of Ki67-positive cells from tissue cores in cancers stratified by molecular subtype. The pairs of cancer subtypes that showed significant difference were indicated by asterisks (*) and arrows (ANOVA at 95% confidence, *p* < 0.01)

Expression levels of biomarkers in single cells in each cored region were evaluated after image analysis of MxIF images and data post-processing. ER and PR histoscores and the percentage of Ki67-positive cells in each cored region were quantified (Fig. 1). There were high levels of heterogeneity in ER and PR histoscores among tissue cored within each individual lumpectomy case (Fig, 1A-B). Dfferences were most prominent in Luminal A cancers for both ER and PR (L56, L69 and L70). Similarly, marked intra-tumoral variations of Ki67 positivity were observed, particularly in the polytypic cancers (Fig. 1C). Comparing ER and PR histoscores per core in cancers stratified based on molecular subtype, basal-like cancers were seen to have significantly lower level of ER histoscore compared to all other cancer subtypes (*p* < 0.01), and lower PR histoscore compared to Luminal A cancers (*p* < 0.01) (Fig. 1D-E). Percentages of Ki67-positive cells in Luminal A cancers were significantly lower compared to Luminal B and HER2-Enriched cancers (*p* < 0.01), while polytypic cancers showed lower Ki67+% compared to Luminal B cancers only (*p* < 0.01) (Fig. 1F). Polytypic cancers also demonstrated broad spectra of ER and PR histoscores (Fig. 1D-E).

**Fig. 2:**
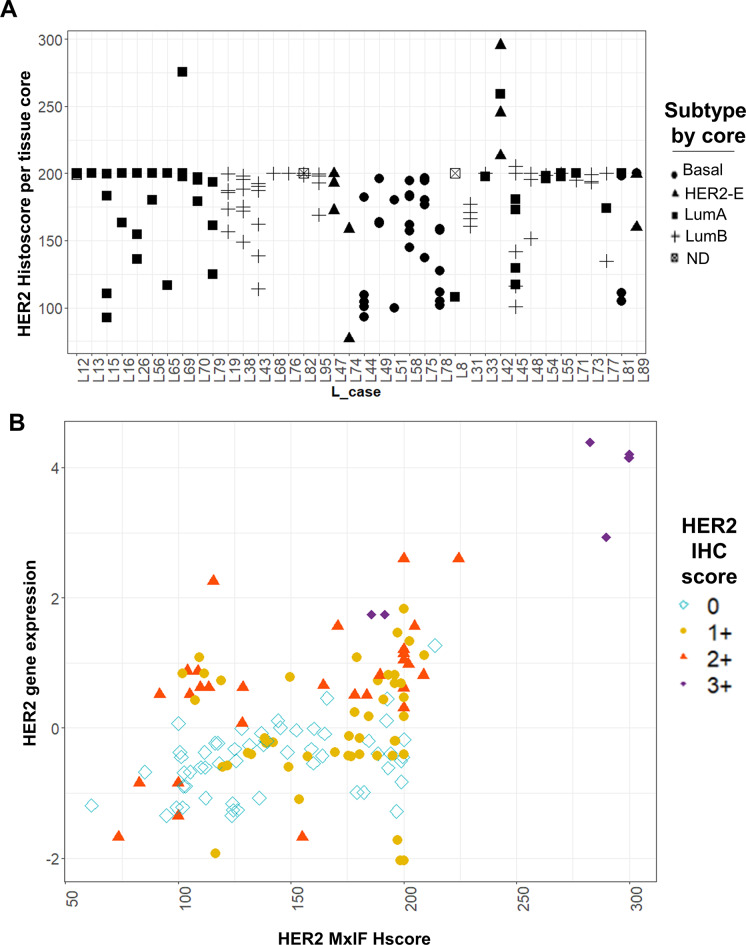
HER2 expression levels measured with MxIF and comparison with HER2 gene expression and clinical IHC scoring. (**A**) HER2 histoscore quantified from MxIF for each cored region per L case was shown. The molecular subtype of each core was represented with the indicated symbol. (**B**) Correlation between the MxIF-quantified HER2 histoscore (Hscore) and HER2 gene expression of the same region. Pearson correlation *R* = 0.59, Spearman correlation *R* = 0.47. The IHC HER2 score from clinical reports for each L case was represented by the colored symbols (0, 1+, 2 + and 3+)

HER2 expression was similarly expressed as histoscore and compared among cored regions of each lumpectomy (Fig. 2). There were variations in the levels of HER2 expression among cored regions from the same cancer (Fig. 2A), however, the only cancers that showed high levels of HER2 were L69 and L42. These two cancers were diagnostically classified as HER2 3 + by IHC (L69 is HR-positive and L42 is HR-negative).

To investigate the relationship among MxIF-measured HER2 histoscore, HER2 mRNA expression measured from tissue core and the clinical HER2 scores determined by pathologists using IHC/ISH, a correlation analysis was performed (Fig. 2B). The overall correlation between HER2 histoscore and HER2 mRNA level per case was low (Pearson correlation *R* = 0.59; Spearman correlation *R* = 0.47). The data point for each tissue core in (Fig. 2B) was further annotated with the HER2 IHC clinical score of the cancer (HER2 0, 1+, 2+, 3+) using colored symbols. We observed that most of the cases scored as 0 by IHC appeared to have consistently lower levels of HER2 mRNA expression. Cases scored as HER2 IHC 1 + and 2 + appeared to have higher HER2 mRNA expression overall, but we failed to observe any strong relationship between HER2 score (1 + vs. 2+) and the respective HER2 mRNA expression levels. Similarly, MxIF-measured histoscore levels were not correlated with IHC HER2 scores of 0, 1 + or 2+. Nevertheless, five of seven cancer cores from HER2 3 + cancers showed high levels of both histoscores and mRNA expression.

### Unsupervised clustering illustrates heterogeneous composition of breast cancer cells

**Fig. 3:**
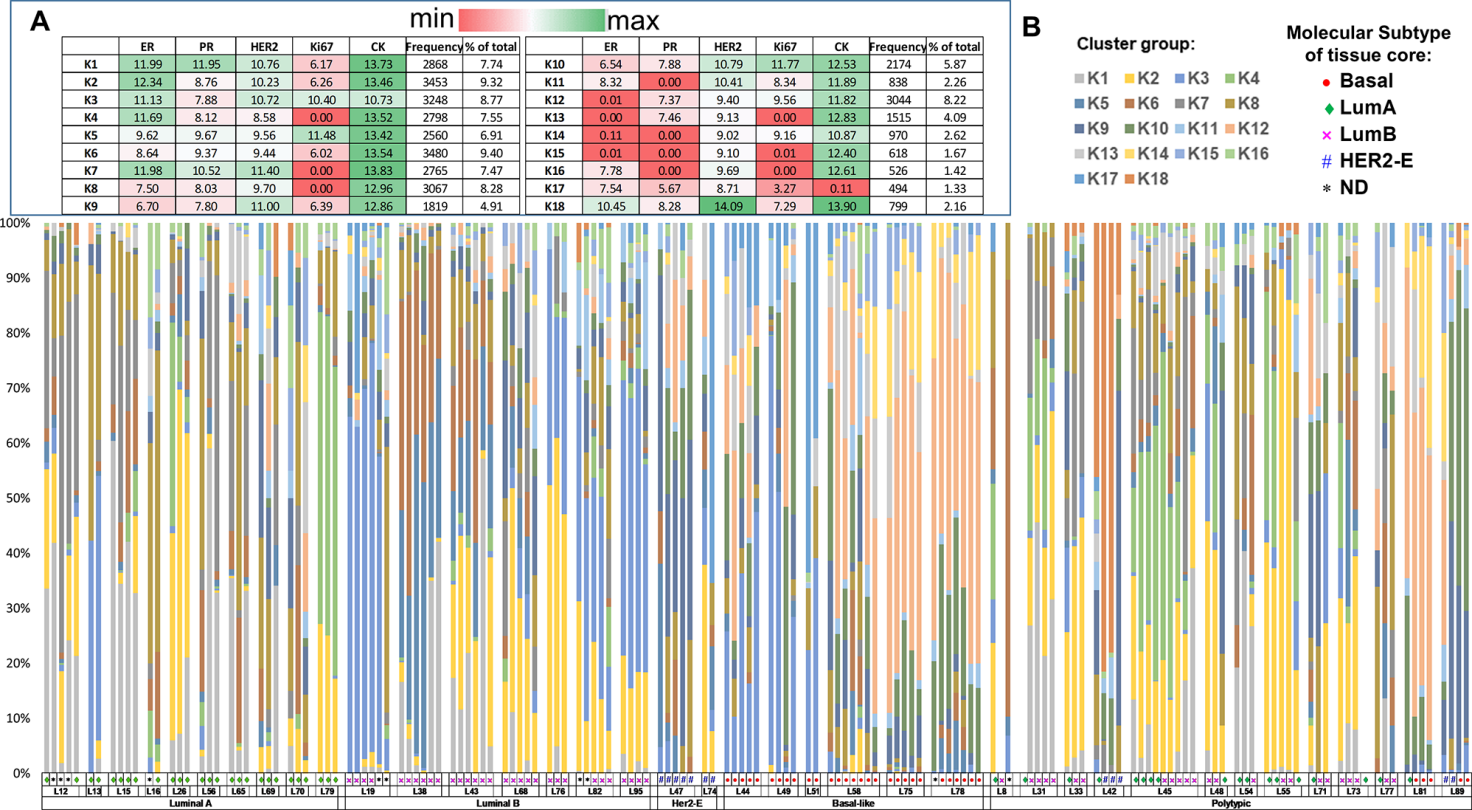
Unsupervised clustering identified cell clusters with similar IHC4 co-expression signatures. (**A**) Table showing the averaged normalised intensity of protein markers in cells classified to each cluster group (K1-K18). The frequency (cell count) of each cluster group and the percentage of total were shown. (**B**) The distribution of cluster groups in each cored region. Cored regions from a lumpectomy (L) case were grouped together. The subtype classification of individual tissue core was identified with the colored symbols in the bottom panel. L cases were stratified according to their overall molecular subtype (Luminal A, Luminal B, HER2-Enriched or Basal-like) or as polytypic cases

To further assess the extent of intra-tumoral heterogeneity in the biomarker co-expression patterns in cancer cells, we used unsupervised clustering to identify cell clusters with similar signatures. A nearest-neighbor graph clustering method (PhenoGraph) [29] conducted with IHC4 protein markers (ER, PR, HER2 and Ki67) and CK intensities yielded 18 groups of cancer cells (K1-18), with a modularity measure of 0.857 (Fig. 3). The averaged intensity level of each protein marker from cells assigned to each cluster group is shown in Fig. 3A. Each cluster group is composed of cancer cells that showed a unique co-expression combination of IHC4 markers. K1-4, 7 and 18 are comprised of cells that have high ER expressions (higher than 10 on average of the normalized expression scale), whereas K12-15 have minimal ER expressions with the remaining groups consisting of low to intermediate ER-expressing cells. As for PR, only two clusters, K1 and 7, showed high levels of expression, and 12 out of 18 cluster groups showed low to intermediate levels (ranged from average of 5.67–9.67) with 4 groups having negative expression levels. Our clustering did not identify a low or negative HER2 group, as all 18 clusters showed intermediate to high levels of HER2 with one group (K18) showing a distinctly high level. For Ki67, almost equal number of cluster groups with high, intermediate and low levels of expression were identified.

The type and percentage of K clusters in each cored region was quantified and compared among regions taken from the same cancer (Fig. 3B). The type of cluster groups present in the cored regions from the same cancer are mostly similar, with a few cases that showed quite distinct patterns among intra-tumoral regions. Among the subtypes of breast cancers, Luminal A cancers appeared to show higher levels of intra-tumoral variations of K cluster types and percentages. A small fraction of the polytypic cases where all cored regions were evaluated showed marked levels of intra-tumoral K clusters variations (L54 and L77 showed the most variations).

### Intra-tumoral heterogeneity in cluster group composition compared among molecular subtypes of breast cancer

**Fig. 4:**
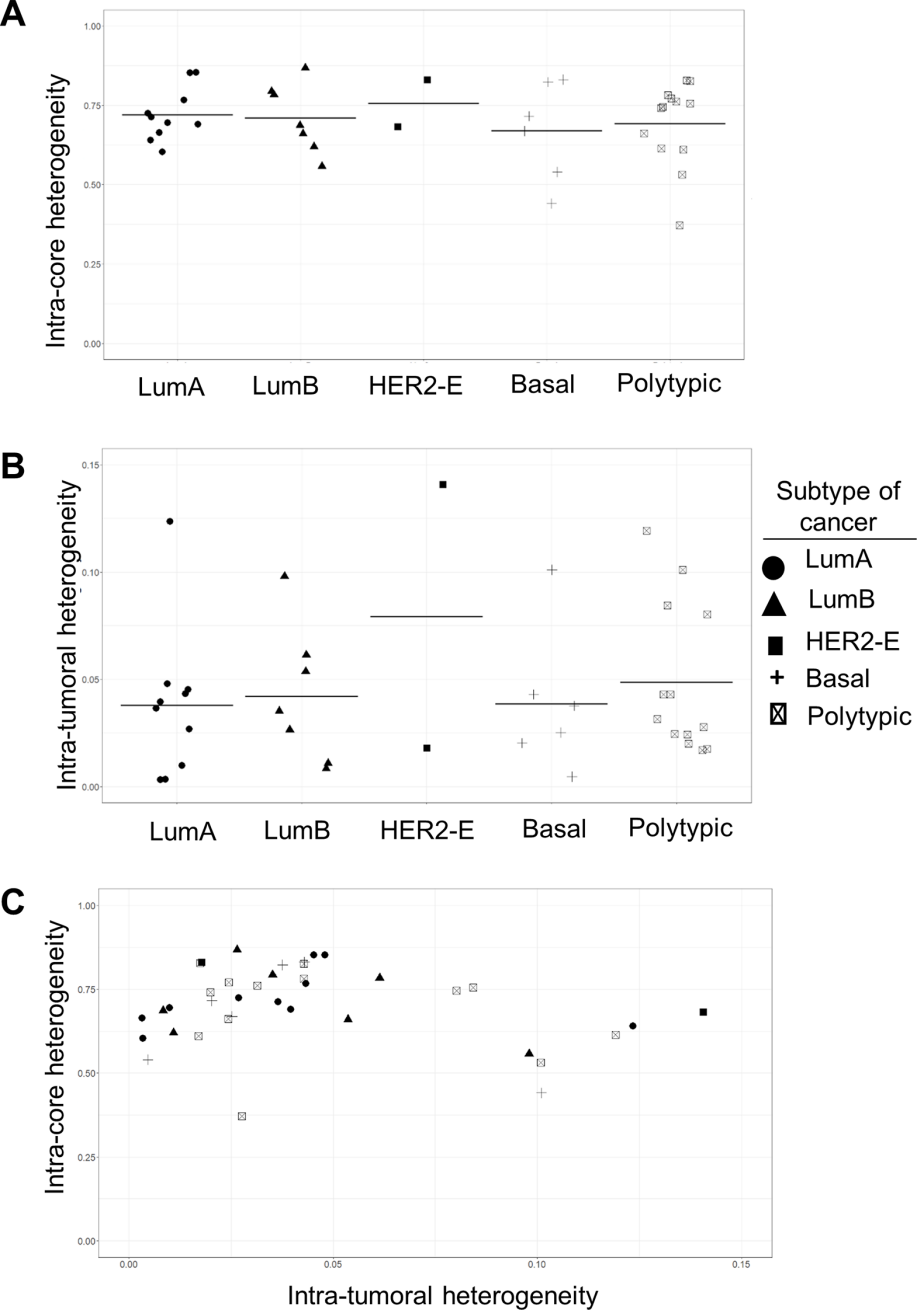
Intra-core and intra-tumoral diversity. A diversity index (Simpson index) based on the presence (type of clusters) and abundance of species (cluster fraction) in a community (tissue core) was used to measure intra-core heterogeneity (within-community diversity) and intra-tumoral heterogeneity (among-community diversity). (**A**) Average intra-core heterogeneity of each lumpectomy case, grouped according to the molecular subtype classification of the cancer (HER2-e, HER2-Enriched). No significant difference detected among subtypes of cancers (ANOVA, 95% confidence level). (**B**) Average intra-tumoral heterogeneity of each lumpectomy case, grouped according to the molecular subtype classification of the cancer. No significant difference detected among subtypes of cancers (ANOVA, 95% confidence level). (**C**) Correlation analysis between intra-core and intra-tumoral heterogeneity for each lumpectomy. Pearson correlation *R*=-0.20 for all samples

We then assessed the extent of intra-core heterogeneity (within each cored region) and intra-tumoral heterogeneity (among the cored regions from a single cancer) of each cancer based on the composition of the 18 K clusters. The Simpson index, a measure of diversity of a community based on the number of species and their abundance, was evaluated for both intra-core and intra-tumor (Fig. 4). Intra-core heterogeneity was quantified as a ratio of the number of clusters present and the proportion of cells assigned to each cluster for each core (Supplemental information: Methods). The degree of intra-core heterogeneity (Fig. 4A) and intra-tumoral heterogeneity (Fig. 4B) were highly similar among subtypes. ANOVA analysis did not detect any significant difference among subtypes for both measurements (95% confidence level). Representative images of tumor cores showing marked level of intra-tumoral diversity, and a cancer case illustrating intra-tumoral diversity in protein expression are shown in Fig. S.3 in Supplemental Information. Intra-tumoral heterogeneity of polytypic cancers was also comparable to lumpectomy cases with a single molecular subtype. To investigate whether the level of heterogeneity within a core is associated with the level of heterogeneity between cores in a single cancer case, a correlation analysis between intra-core heterogeneity and intra-tumoral heterogeneity was conducted (Fig. 4C). Over the entire range of intra-tumoral heterogeneity only a weak negative association (Pearson *R*=-0.20) was observed, whereas over the lower end of the range there was a slight positive correlation (Pearson *R* = 0.45).

### Image patch analysis to identify spatial immune features

**Fig. 5:**
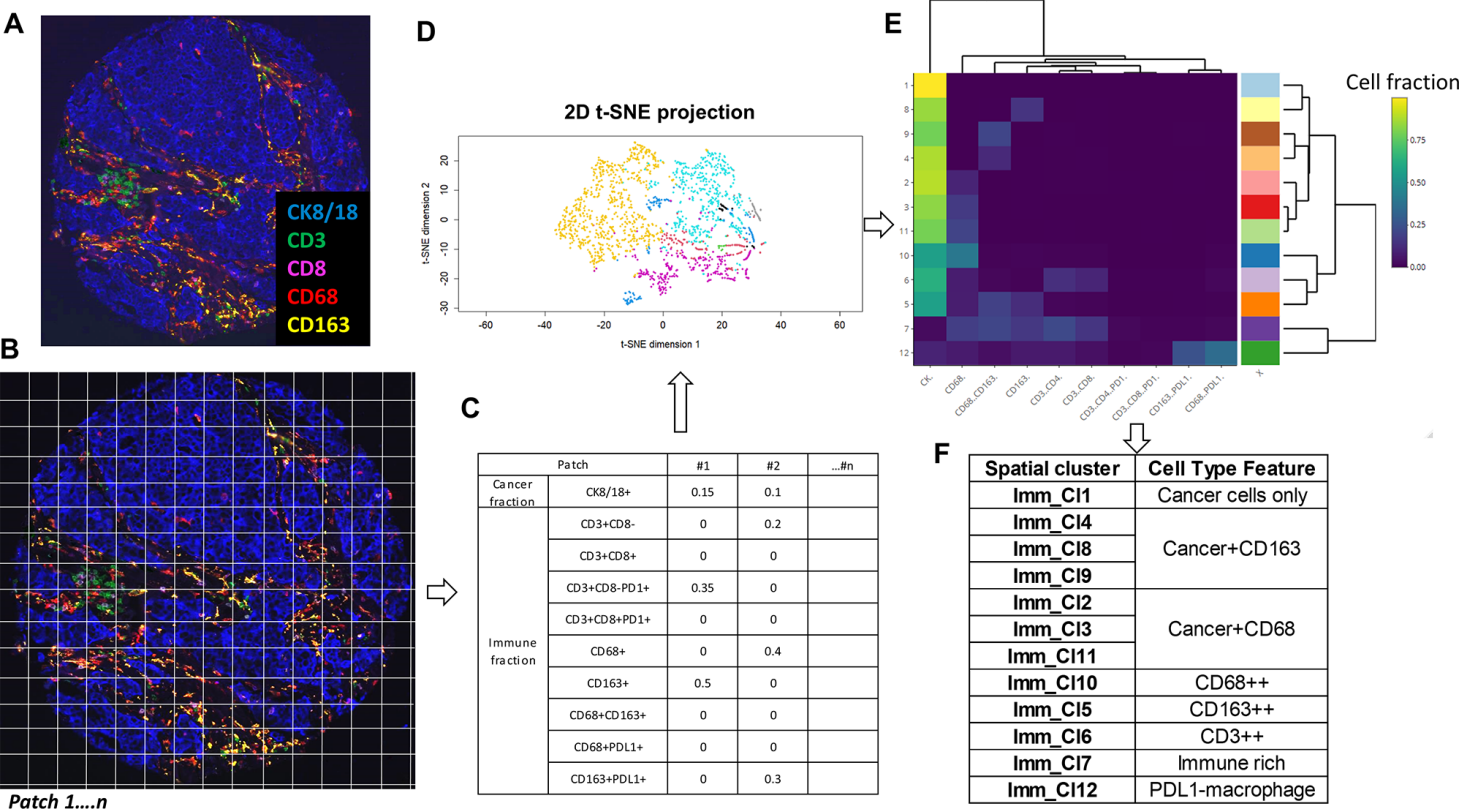
A schematic illustrating image patch analysis of immune spatial phenotypes. (**A**-**B**) A representative MxIF image was divided into small patches (150 pixel in dimension). (**C**-**D**) The fraction of each immune subset and cancer cell fraction (CK) in each patch were used in unsupervised clustering. (**E**) A total of 12 spatial clusters (Imm_Cl1-12) was identified, and a dendrogram was conducted to identify clusters that were similar in their cancer/immune composition. (**F**) Grouping of similar Imm_Cl identified 8 unique immune spatial phenotypes

In addition to investigating heterogeneity in IHC4 biomarker expression in the cancer epithelium, we sought to determine the extent of heterogeneity in the spatial localizations of immune cells. Quantitative image analysis of multiplexed protein staining of immune lineage markers was performed in a separate immunofluorescence multiplex staining study with antibodies labelling various immune cell subsets (Fig. 5). Each image of a TMA core was divided into image patches of 150 pixel x 150 pixel (48.75 μm x 48.75 μm) (Fig. 5A-B), and the fraction of cells in each immune cell type, together with the cancer cell fraction (CK+) were quantified for each patch (Fig. 5C). Patches containing fewer than 5 cells were excluded from analysis as were TMA cores with fewer than 10 patches identified (due to tissue damage or poor cellularity).

A total of 4895 patches from 22 lumpectomy cases, with an average cell count of 9 per patch (median = 8) was included in an unsupervised clustering with PhenoGraph to identify patterns of immune phenotype (Fig. 5D). A total of 12 “spatial immune clusters” was generated. Examination of the spatial clusters showed various combinations of immune and cancer cell densities. A dendrogram illustrated the similarities between clusters (Fig. 5E). Subsequently, clusters which showed a similar pattern of cell densities were aggregated and a unique cell type feature for each grouped cluster was identified (Fig. 5F). Eight distinct cell type features were identified, ranging from immune “cold” phenotypes of high cancer cell proportions and low immune cell proportions (e.g., feature with only cancer cells; cancer with few CD163 + cells; cancer with few CD68 + cells); to immune “hot” phenotypes with a majority of immune cells present (e.g., CD68-rich (CD68++), CD163-rich (CD163++), T lymphocyte-rich (CD3++), overall immune cell-rich (Immune rich)) and a group with high PDL1 expression on macrophages (PDL1-macrophage).

### Spatial heterogeneity in the immune microenvironment

**Fig. 6:**
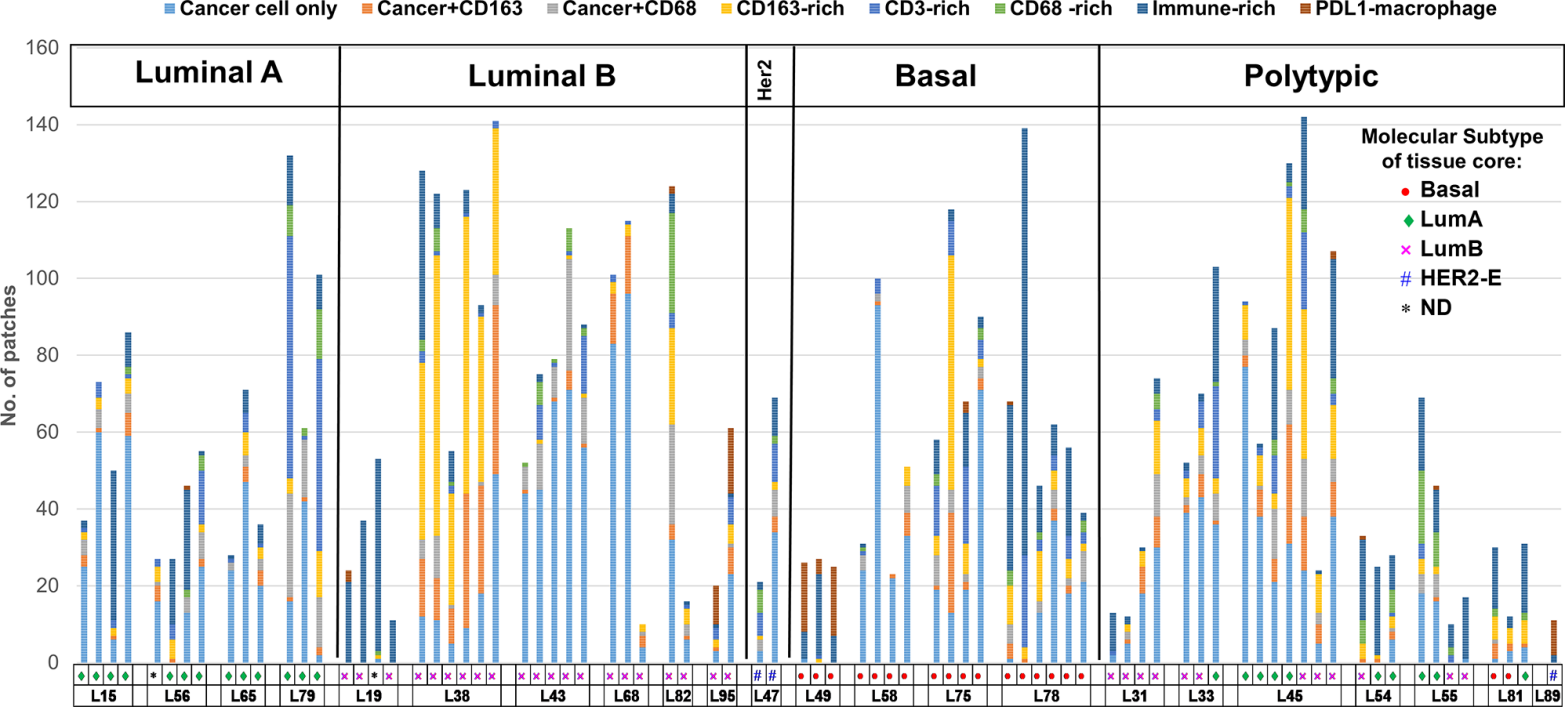
Immune spatial phenotypes in breast cancer. The distribution of immune spatial phenotypes in each cored region was shown. The molecular subtype classification of each tissue core was identified with the indicated color symbols in the bottom panel. Cancer cases were grouped according to their overall molecular subtype (Luminal A, Luminal B, HER2-Enriched, Basal-like, Polytypic)

**Fig. 7:**
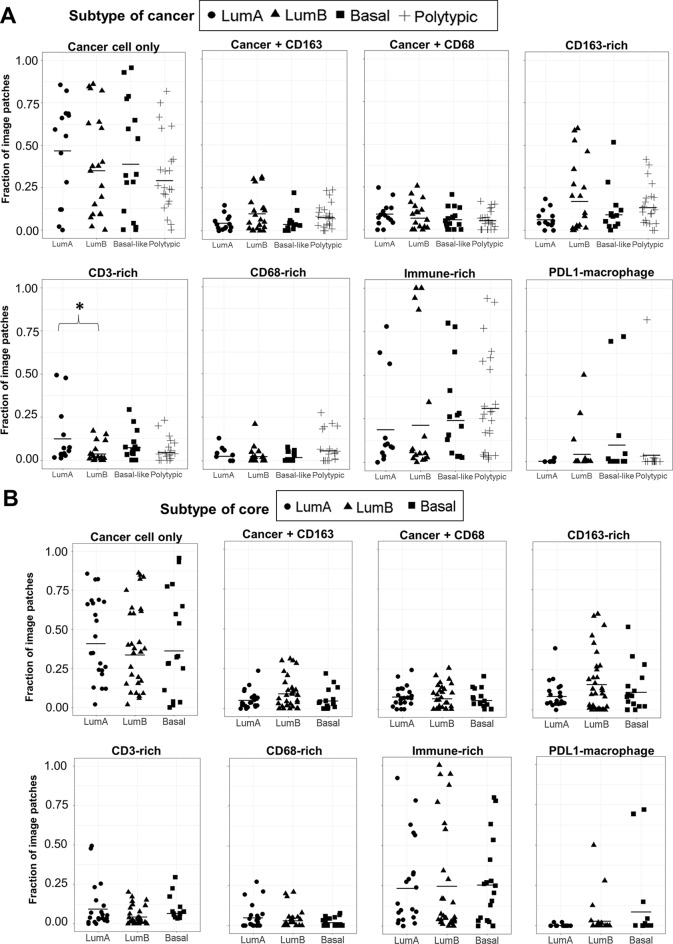
Comparison of immune spatial phenotypes among breast cancer subtypes. (**A**) The fraction of image patches in each tumor core that showed the indicated spatial feature was plotted, compared among cancers of various molecular subtype (LumA, LumB, basal-like and polytypic). *N* = 14 cores in LumA cancers, *N* = 22 cores in LumB cancers, *N* = 17 cores in basal-like cancers, *N* = 25 cores in polytypic cancers. There were 2 tissue cores in one HER2-enriched cancer and are excluded from the analysis. Tissue cores in Luminal A cancers were found to show more CD3-rich features when compared to cores in Luminal B cancers (ANOVA, *p* = 0.036). (**B**) The fraction of image patches in each tumor core that showed the indicated spatial feature compared among the molecular subtypes of the tissue core. *N* = 24 for LumA, *N* = 34 for LumB and *N* = 19 for basal-like cores. There were only 3 HER2-enriched cores and therefore excluded from this analysis. No significant difference were found between subtypes. The scale on the y-axis is the same for all panels (0.00–1.00)

The distribution of immune spatial features observed in each analysed cored region is shown in Fig. 6. Cored regions taken from a single lumpectomy are arranged together for comparison. While most analysed regions from an individual cancer exhibited similar levels of cancer (CK+) and immune spatial clusters, Luminal A cancers appeared to show a more diversified cancer/immune content, particularly cases of L15, L56 and L79. For each immune spatial feature, we measured the fraction of image patches in each tissue core assigned to that feature and compared among cancers stratified by their molecular subtypes (Fig. 7A). Luminal A cancers were found to show higher levels of CD3-rich features compared to Luminal B cancers (ANOVA analysis, *p* = 0.036).

We then measured the frequencies of each spatial immune feature in tissue cores and compared among molecular subtypes of the core to investigate whether the immune phenotype is correlated with the molecular profile of the tissue core itself (Fig. 7B). While CD163-rich patches were seen at a higher level in LumB cores, we did not find any significant difference between molecular subtypes of the core for any features (ANOVA analysis at 95% confidence level). HER2-enriched tissue cores were not included as the sample size is extremely small (*n* = 3).

To determine whether the immune phenotype is altered in polytypic cancers, we further compared the immune phenotype of LumA and LumB cores in polytypic cancers only. LumA and LumB cores were the most common subtype combinations in polytypic cancers (10 out of 13 polytypic cancers were comprised of mixed LumA and LumB cores). We found that both subtypes appeared to have high frequencies of immune-rich patches (Fig. S.8). However, there was no significant difference detected for any immune feature between LumA and LumB cores (Wilcoxon rank sum test).

## Discussion

Spatial heterogeneity is a well noted feature of breast cancer. Using immunofluorescence protein multiplexing of a collection of whole-mount processed lumpectomies, we have assessed the level of variations in protein expression and immune phenotype within individual breast cancer lesion. Interestingly, a subset of cases demonstrates multiple subtype assignments within an individual cancer. We have assessed the range of IHC4 (Estrogen Receptor (ER), Progesterone Receptor (PR), HER2 and Ki67) protein marker expression and compared the Histoscores across regions of the same lumpectomy. We studied HER2 protein expression levels in single cells and compared the intra-tumoral spectrum to HER2 gene expression and HER2 scoring based on IHC evaluation. Unsupervised clustering identified cluster groups of cells that exhibit various co-expression signatures, further demonstrating the heterogeneous nature between regions within a single cancer. Furthermore, we applied image analysis with single cell phenotyping to reveal niches of immune phenotype and found that some regions showed higher immune densities compared to other intra-tumoral areas. Our findings of the extent of intra-tumoral heterogeneity in breast cancer suggest its potential impact on diagnosis and subsequent therapy planning.

Variations in the expression levels of hormonal receptors ER and PR are frequently observed. Typically, the clinical pathologic evaluation of biomarkers results in a single measurement ostensibly representative of the entire lesion, characterising expression as weak, moderate or strongly stained, accompanied by an estimate of the percentage of cancer cells positive for the biomarker. Cancers with > 1% positivity for ER or PR are considered as hormone receptor-positive cancer and treated with endocrine therapy [36, 37]. However, with the wide intra-tumoral variability of biomarker staining intensity and % positivity, it remains unclear which breast cancers will respond best to hormonal therapy, and which ones should be treated with alternative or additional regimens. USCAP guidelines for hormonal receptor testing in breast cancer now recommend the report of cancers that have low (1–10%) ER-positivity to distinguish from those with higher ER expression levels in recognition of the limited data on responsiveness of the former group to endocrine therapy [36].

Recently, the expression level of HER2 in pathologically classified HER2-low (HER2 IHC 1 + or 2+, ISH non-amplified) cancers has been a topic of discussion after the DESTINY-Breast04 trial showed positive results in the treatment of HER2-low metastatic cancers with HER2-targeting antibody-drug conjugates (ADC) [38]. This raises the question of how much HER2 protein is being expressed in these HER2-low cancers that are responding to ADCs and the mechanism of the response. It also pointed to other possibilities of treating primary HER2-low cancers with ADC. Our investigation suggests that there is a wide range of HER2 protein expression levels in individual cells quantified with our technology and failed to show a strong correlation to IHC score (1 + or 2+) of the whole lesion. This failure could be due to inadequate sensitivity of our platform in detecting low-expressing cells, or to the fact that while protein multiplexing provides an averaged quantification of HER2 protein expression of the cell, the ASCO-CAP guidelines [39] for IHC scoring considers not only expression but also the morphology of staining (e.g. complete membranous vs. partial membranous staining). For IHC HER2 “0” cancers, a majority of cases showed consistently low levels of HER2 gene expression, while protein levels quantified with MxIF spread over a wide spectrum. Others have also demonstrated a discordance between HER2 IHC and ERBB2 expression [40], or failed to correlate HER2 protein or RNA with clinical HER2 “0”, ultra-low or 1 + cases [41]. Studies have also shown that HER2-low cancers with higher HER2 expression (HER2 2+) did not confer a benefit in response to HER2-ADC compared to HER2 1+ [38, 42]. This raises the question of whether IHC scoring adequately stratifies HER2 expression levels, or if there are other factors that contribute to HER2-targeted drug delivery, and both scenarios would require further studies. Comparison among TMA cores taken across the whole lumpectomy revealed that some cores showed much higher levels of HER2 staining than other cores, suggesting that HER2 intensity levels varied spatially as other reports have shown [43–46], and the resulting scores may differ depending on the precise location of biopsy sampling.

While most of the cores taken from a lumpectomy showed a single, consistent molecular subtype, 13 out of 38 cases analysed showed multiple molecular subtypes. This observation highlights both intra-tumoral spatial as well as cellular heterogeneity of biomarker expression. Tumor heterogeneity may be implicated in the recurrence of the disease after treatment of the primary lesion. It has been reported that up to 36% of metastatic breast cancer showed a “switch” of the cancer subtype from primary diagnosis [47]. Whether this is a true event of the adaptive response of the cancer to treatment, or it simply is a result of tumor heterogeneity remains to be investigated. The frequency of molecular-polytypic cancer in our small study (13 out of 38, or 34%) suggests that this incidence could be higher than expected, however, we note that this cohort was enriched for multifocal cancers. Intra-tumoral heterogeneity is a result of clonal evolution [5, 9–12, 48]. The presence of breast cancer stem cells, cells that possess stem-like properties which can self-renew and give rise to all cell types in a cancer has also been suggested as an underlying mechanism of intra-tumoral heterogeneity [49–51]. The presence of multiple molecular subtype lesions impose significant impact on treatment strategies. Using combination therapies instead of a single line of treatment could maximize the chances that all cancer cells are targeted [4]. As the biomarker status of many breast cancers were evaluated on core biopsy, our study suggests that a more comprehensive sampling of the breast cancer for biomarker status confirmation could be beneficial. While most studies reported the average number of core needle biopsies sufficient for diagnosis of a cancerous lesion ranged from 3 to 5 [52–54], this could be sufficient for smaller tumors with larger tumors requiring more than 5 core biopsies to capture biomarker and spatial heterogeneity.

Outcome analysis of our cohort at 10 year follow-up showed that there were 8 recurrences (out of 38 cases) (Table 1). Unfortunately, all recurrences were metastatic and lethal. We noted that 3 out of 8 recurrences/death were polytypic cancers. Whether the presence of polytypic lesions resulted in more aggressive cancers warrant further investigations.

Results from our study also suggested the benefits of using whole-mount (WM) histopathology techniques for processing some of the larger, more advanced breast tumors. Our lab and others have reported on the advantages of WM techniques (also known as large-format histology) in terms of improved assessment of margins, focality and volume estimates of breast cancer [20, 21, 55–58]. Costing analyses suggested that WM techniques are more costly compared to standard histo-processing but offer advantages such as improved assessment of tumor heterogeneity and correlation to radiological findings [59, 60]. Ultimately this could result in fewer recurrences and an improvement in quality-adjusted life years (QALYs) [61]. We believe that selective adoption of WM techniques in clinical pathology facilities, focusing on tumors that are larger, more advanced, or from women with dense breasts (breast with high degree of fibro-glandular composition) where in vivo imaging could be inadequate in providing comprehensive examinations of the entire breast, could provide significant impact on breast cancer diagnosis and treatment.

Spatial heterogeneity of cells in the immune microenvironment has also been studied widely with high-dimensional single cell phenotyping technologies, specifically in TNBC, given that immune checkpoint inhibitors (ICI) targeting programmed death-1 (PD-1) (anti-PD1 pembrolizumab) plus chemotherapy [62] was approved for TNBC. Nevertheless, not all TNBC cases respond to pembrolizumab [63], and research has discovered cellular and spatial signatures predicting response in TNBC [64]. TNBC and HER2-positive (hormonal receptor-negative) cancers are known to harbor higher levels of tumor-infiltrating lymphocytes compared to hormonal receptor-positive cancers [65]. Increased levels of tumor infiltrating lymphocytes (TILs) could predict response to neoadjuvant chemotherapy in breast cancer [66, 67] and is also associated with better outcomes of ER-negative/HER2-negative cancers [68–71]. In the current report, we used a quantitative approach combining immune densities and spatial localization to study the identity of immune subsets in each tissue core and found that most cores studied from the same cancer showed a similar pattern of cancer/immune composition. We demonstrated that Luminal A cancers, which have an overall lower immune cell count, appeared to show a slightly more heterogeneous makeup of immune lineage cells and arrangements. Statistical analysis showed that Luminal A cancers showed a higher level of CD3-rich features compared to Luminal B cancers. Our analysis did not show higher levels of immune features present in basal-like cancers unlike what other reports have shown [65, 67, 70, 71]. The fact that our study was limited to small regions of the cancer has prohibited us from conducting a more comprehensive assessment of the tumor microenvironment. Although multiple regions were extracted from whole-mount processed samples, many of the cores were taken from areas of dense cancer cellularity instead of the stroma compartment where some of these tertiary lymphoid structures, composed of clusters of immune cells known to positively associate with prognosis, may be localized [72].

More in-depth examination is required to gain an understanding of the underlying drivers causing heterogeneity. Efforts on revealing spatial heterogeneity in solid cancers with various spatial single cell transcriptomic and proteomic technologies have unveiled quantitative features from cluster signatures, cellular morphology to architectural subtypes for better characterization of disease [64, 73–76]. One common theme that has emerged is that cancer is an “ecosystem” composed not only of cancer cells, but also their interaction with immune cells, cancer-associated fibroblasts and vascular cells in the tumor microenvironment. In addition to single-cell RNA and protein expression profiling, recent studies in polygenic risks have suggested that up to 18% of familial heritability of breast cancers could be attributed to a large number of low-risk genetic variants, such as single nucleotide polymorphisms (SNPs) [77]. Each variant explains a small proportion of risk of the disease, but in combination their presence could predict a significant risk. A large number of these SNPs have been identified as having a role in determining molecular subtype or breast cancer risk [78–80]. It is not known how these polygenic factors could also contribute to the protein expression and cellular heterogeneity that we and others have observed, and this would warrant further investigations.

## Conclusions

Our study highlights the high degree of biomarker and spatial intra-tumoral heterogeneity, supporting the need for more comprehensive sampling and evaluation of breast cancer for diagnosis and treatment planning.

## Electronic supplementary material

Below is the link to the electronic supplementary material.

Supplementary Material 1

## Data Availability

The datasets used and analysed during the current study are available from the corresponding author on reasonable request.

## Abbreviations

BIRL: Biomarker Imaging Research Laboratory (Sunnybrook Research Institute, ON, Canada)
ER: Estrogen Receptor
FFPE: Formalin-fixed, paraffin-embedded
GINA: *Genomic-Imaging Neo-pathologic Analysis cohort*
HER2: Epidermal Growth Factor Receptor 2
H&E: Haematoxylin & Eosin
ICI: Immune checkpoint inhibitors
IHC: Immunohistochemistry
ISH: *In Situ* Hybridization
MxIF: Multiplex in Immunofluorescence
PR: Progesterone Receptor
REB: Research Ethics Board
TMA: Tissue microarray
TNBC: Triple-negative breast cancer
