# Supplementary material for: Intra-tumoral spatial heterogeneity in breast cancer quantified using high-dimensional protein multiplexing and single cell phenotyping
Source: Breast Cancer Res. 2025 May 21;27:88. doi: 10.1186/s13058-025-02038-1 (PMC12096620; doi:10.1186/s13058-025-02038-1)
Supplement: Supplementary file 1 — Supplementary Material 1 [file 13058_2025_2038_MOESM1_ESM.pdf]

Intra-tumoral spatial heterogeneity in breast cancer quantified using high-dimensional protein multiplexing and single cell phenotyping

Cheung AM, Wang D, Quintayo MA, Yerofeyeva Y, Spears M, Bartlett JMS, Stein L, Bayani J, and Yaffe MJ

Supplemental Information

Table of Contents

Methods:

- Definition of multi-focal cancers
- Antibody Staining methods
  - Protein multiplexing
  - Immunohistochemical staining
  - HER2 IHC (clinical)
  - HER2 In situ hybridization (clinical)
- PhenoGraph and clustering parameters
- Diversity metrics

Tables:

- Table S.I- List of antibodies used in MxIF and IHC, suppliers, clones and concentrations; order of antibody staining of IHC4 panel and Immune panel
- Table S.II – List of all lumpectomy cases included in our study: IHC subtype (case), and molecular subtype (core and case)

Figures:

- Fig. S.1- Schematic diagram illustrating the workflow of our study
- Fig. S.2 - Representative examples illustrating the selection of cut-levels of normalised expression of ER and PR for 1+, 2+ and 3+ categories
- Fig. S.3 – Representative MxIF images from intra-tumoral images of a breast cancer molecularly subtyped as Luminal A, including examples of intra-core and intra-tumoral heterogeneity
- Fig. S.4 - Representative MxIF images from intra-tumoral images of a breast cancer molecularly subtyped as Luminal B.

Fig. S.5 - Representative MxIF images from intra-tumoral images of two (IHC-scored) HER2 3+ breast cancers.

Fig. S.6 - Representative MxIF images from intra-tumoral images of a basal-like breast cancer.

Fig. S.7 - Representative MxIF images from intra-tumoral images of two poly-typic breast cancers.

Fig. S.8 – Comparison of immune phenotype between LumA vs LumB cores in polytypic cancers.

References

Definition of multi-focal cancers:

In our dataset distinct foci are at least 4 mm apart. In our whole-mount tissue processing protocol, the specimens were sectioned into 4 mm slices using a deli slicer (Clarke et al., 2013. Each slice was processed, paraffin-embedded and sectioned at 4 μm. Cancers were considered to be multi-focal if 1) they resided within the same tissue block and distinct foci were measured as being at least 4mm apart, 2) they were found on adjacent tissue blocks at spatially distinct (at least 4mm apart) x-y locations, 3) in approximately the same x-y location on blocks separated by at least one block (in z axis) where cancer was not observed.

Antibody staining methods:

Protein multiplexing:

Before staining, tissue sections were dewaxed in xylene and rehydrated in graded ethanol and distilled water. After 5-minutes of washing with phosphate buffered saline (PBS), antigen retrieval was done using a pressurized cooking chamber (Decloaking Chamber Plus™; Biocare Medical, Concord, CA). Within a pressurized chamber, the following sequence was applied to each section: (i) heating to 110°C in citrate buffer (ii) 4 min incubation in citrate (iii) discontinuation of heating (iv) 16 min in citrate; and (v) 20 min in Tris EDTA buffer. The slides were then removed for passive cooling to room temperature. Tris EDTA buffer (pH 9.0) was found to be a necessary adjunct to citrate buffer (pH 6.0) to accommodate the pH requirements of different epitopes simultaneously while avoiding any complication associated with over-retrieval (e.g. tissue loss) (Clarke et.al., 2016 Histopathology). The slides were then blocked with a background blocking reagent (Background Sniper; Biocare Medical, Concord, CA) for 15 minutes and counterstained for 15 minutes with DAPI (4'6-diamidino-2-phenylindole) (Invitrogen™, Thermo Fisher Scientific, Waltham, USA) for nuclear identification and for image registration (Wang et al., 2024). Proceed with imaging on the protein multiplexing system.

Immunohistochemical staining (Large slides):

Tissue sections were deparaffinized in Xylene and rehydrated. For antigen retrieval, slides were immersed in the staining jar containing Antigen Retrieval Buffer (Biocare Medical). The staining jar was then placed in the pressure cooking chamber with 500ml of water. Preheat at 70°C for 20 minutes, incubate at 110°C for 4 minutes, then cool down to 90°C. Remove the staining jar to room temperature and allow the slides to cool for 20 minutes. Slides were incubated in 3% H_2O_2 in distilled water for 10 minutes at room temperature for peroxidase blocking. Incubation with primary antibodies were conducted according to the protocol (see Table S.I). Secondary antibodies from MACH 3 (Biocare Medical) were incubated according to manufacturers' protocol. Chromogen detection was conducted with MACH 3 HRP Polymer Detection (Biocare Medical) followed with DAB solution (DAKO), according to manufacturers' protocols. Stained sections were then counterstained with hematoxylin for 30 seconds, dehydrated with ethanol and mounted with coverslips.

HER2 Immunohistochemical staining (Department of Anatomic Pathology, Sunnybrook Health Sciences Center):

Staining was conducted with VENTANA anti-HER2/neu (4B5, Roche Diagnostics) following manufacturers' protocol and following the guidelines from USCAP (Wolff AC et al., 2018, Bartley AN et al., 2017).

HER2 In situ hybridization (Department of Anatomic Pathology, Sunnybrook Health Sciences Centre) (Wolff AC et al., 2018; Bartley AN et al., 2017):

Slides are removed from rapid microwave and rinse with Tris buffer. 1-3 drops of fresh, stable pepsin at room temperature are added directly to the center of the tissue section. Wash with distilled water and dehydrate with 70% ethanol. Apply 5-10 ul of HER2/neu/CEP probe solution (Vysis FISH kit, Abbott Molecular) to the center of the tissue section. Apply coverslip to the tissue section and sealed. Place the slides into a Hybrite Incubator. Co-denaturation of the tissue sections occurs at 73°C for 10 minutes, followed by hybridization at 37°C for at least 18 hours. Remove coverslips and immerse slides in preheated, post-hybridization wash buffer at 73°C for 2 minutes. Slides are then agitated in distilled water for 2 minutes, and allow to air dry in the dark for 1 hour. Apply 5-10 ul of DAPI counterstain (Vysis kit) to the center of the tissue section and apply coverslip. Check quality of staining under the fluorescent microscope.

Phenograph and clustering parameters:

Unsupervised clustering for cancer epithelial cells (CK, ER, PR, HER2, Ki67) (Figure 3) and for immune patch phenotypes (Figure 5) were conducted using the published algorithm PhenoGraph (Levine JH et al., Cell 162(1):184-197, 2015). This method is a graph-based k-nearest neighbor clustering algorithm, with a user-defined value of k. We selected this method for our clustering analysis as the algorithm analyses high-dimensional single cell data by first creating graph-based networks of cells with similar signatures followed by clustering into phenotypic communities. The range of values of k for testing was determined based on the size of the cell population. The total number (n) of cancer cells were 37,031, and the total number of patches analysed for immune phenotype (n) was 4,895. Clustering of the cancer population started with k=200, in order to obtain a reasonable number of clusters (25-40) that are meaningful representative of the population that would allow us to study the composition of these clusters in each cancer case in a practical manner. Similarly with immune patches, we started testing at a much lower k since n was much lower. The modularity, a measure to describe the "correctness" (in an energy-minimization sense) provided us with the quality of each clustering output. The value of modularity ranges from -1 to 1 and the higher the value the better the quality of the graphs/cluster assignments. The optimal number of clusters was determined by testing different values of k and maximizing the value of modularity.

Diversity metrics:

Heterogeneity of K cluster groups in cored regions and cancer are measured using function raoD in R statistics (package "picante"). raoD outputs quadratic entropy, which measures diversity based on the type and abundance of species in a community, taking into account the dissimilarity of species (Rao . In this study, we do not treat clusters as dissimilar and therefore no dissimilarity index was applied. As a result, raoD's diversity output is equivalent to Simpson index. In the output, raoD measures Dkk, which is the probability that two individuals drawn from a community are from different taxa (within-community diversity). We use this metric as a measure of intra-core heterogeneity (probability that two cells from the same core belong to different K cluster groups). Another output from raoD is Dkl, a beta-diversity equivalent of

Simpson's diversity. This is an estimate of the probability that two individuals drawn from each of two communities belong to different taxa (among-community diversity). We use this metric as a measure of intra-tumoral heterogeneity (probability that two cells from two cores belong to different K cluster groups) (Rao CR, 1982; Jost L, 2007).

IHC4 panel

Round #	Cy3	Supplier/Vendor (clone)	Concentration (ug/ml or dilution)	Cy5	Supplier/Vendor (clone)	Concentration (ug/ml or dilution)
1	PR	Dako (PR1294)	20	HER2	Roche (4B5)	(1:100)
2	NaKATPase	Abcam (EP1845Y)	8	ER	Spring Biosciences (SP1)	5
3	CK8/18	Novex life tech (Zym5.2)	10	Ki67	Zeta (SP6)	10
4	Ribosomal S6	Cell Signaling (5G10)	5			

Immune panel

Round #	Cy3	Supplier/Vendor (clone)	Concentration (ug/ml or dilution)	Cy5	Supplier/Vendor (clone)	Concentration (ug/ml or dilution)
1	PDL1	Roche (SP263)	(1:100)	CD8	Dako (C8/144B)	2.5
2	NaKATPase	Abcam (EP1845Y)	8	PD1	Abcam (EPR4877(2))	20
3	CK8/18	Novex life tech (Zym5.2)	10	CD3	Dako (F7.2.38)	5
4	Ribosomal S6	Cell Signaling (5G10)	5	Ki67	Zeta (SP6)	10
5	CD163	BioRad (EDHU-1)	10	FoxP3	BioLegend (150D)	(1:50)
6				CD68	ThermoFisher(KP-1)	5

WM IHC

	Species	Supplier	Catelog No.	Dilution	Incubation time
ER	Mouse Monoclonal	Leica Biosystems	NCL-ER-6F11	1:50	1 hr at room temperature
PR	Mouse Monoclonal	Leica Biosystems	NCL-PGR-312	1:50	1 hr at room temperature

Table S.1: List of antibodies used in MxIF and WM IHC, suppliers, clones and concentrations; order of antibody staining for MxIF IHC4 panel and Immune panel

L case	IHC-classification	tissue core	Molecular Subtype by core	Molecular Subtype by case
L12	HR+, Her2 0	B2	Luminal A	Luminal A
		B10	ND	
		B11	Luminal A	
L13	HR+, Her2 ND (DCIS)	B6	Luminal A	Luminal A
		B9	Luminal A	
L15	HR+, Her2 0	B13_1	Luminal A	Luminal A
		B13_2	Luminal A	
L16	HR+, Her2 1+	B6	ND	Luminal A
		B9	Luminal A	
L26	HR+, Her2 0	B11_1	Luminal A	Luminal A
		B11_2	Luminal A	
		B8	Luminal A	
L56	HR+, Her2 1+	B7	Luminal A	Luminal A
		B8	Luminal A	
		B9	Luminal A	
L65	HR+, Her2 0	B12	Luminal A	Luminal A
		B13	Luminal A	
		B14	Luminal A	
L69	HR+, Her2 3+	B8	Luminal A	Luminal A
		B10	Luminal A	
L70	HR+, Her2 1+	B11	Luminal A	Luminal A
		B14	Luminal A	
		B17	Luminal A	
L79	HR+, Her2 1+	B7_1	Luminal A	Luminal A
		B7_2	Luminal A	
L19	HR+, Her2 1+	B6	Luminal B	Luminal B
		B7_1	ND	
		B7_2	Luminal B	
		B7_3	ND	
L38	HR+, Her2 0	B6	Luminal B	Luminal B
		B7_1	Luminal B	
		B7_2	Luminal B	
		B11	Luminal B	
L43	HR+, Her2 0	B7	Luminal B	Luminal B
		B9	Luminal B	
		B10	Luminal B	
L68	HR+, Her2 2+ (Not amplified	B22_1	Luminal B	Luminal B
		B22_2	Luminal B	
		B24	Luminal B	
L76	HR+, Her2 1+	B5	Luminal B	Luminal B
		B7	Luminal B	
		B9	Luminal B	
L82	HR+, Her2 1+	B7	ND	Luminal B
		B8	Luminal B	
		B9	Luminal B	
L95	HR+, Her2 1+	B8	Luminal B	Luminal B
		B10	Luminal B	

		B12	Luminal B	
L47	HR-, HER2 3+	B4	HER2-Enriched	HER2-Enriched
		B7	HER2-Enriched	
		B9	HER2-Enriched	
L74	HR+, Her2 0	B8_1	HER2-Enriched	HER2-Enriched
		B8_2	HER2-Enriched	
		B10_1	HER2-Enriched	
		B10_2	HER2-Enriched	
		B11_1	HER2-Enriched	
		B11_2	HER2-Enriched	
L44	TNBC	B11	Basal-like	Basal-like
		B12	Basal-like	
		B13	Basal-like	
L49		B8	Basal-like	Basal-like
		B10	Basal-like	
		B11	Basal-like	
L51	TNBC	B22	Basal-like	Basal-like
		B25	Basal-like	
		B31_1	Basal-like	
		B31_2	Basal-like	
L58		B6_1	Basal-like	Basal-like
		B6_2	Basal-like	
		B7_1	Basal-like	
		B7_2	Basal-like	
		B8_1	Basal-like	
		B8_2	Basal-like	
L75	TNBC	B4	Basal-like	Basal-like
		B6	Basal-like	
		B7	Basal-like	
L78	TNBC	B7_1	ND	Basal-like
		B7_2	ND	
		B9_1	Basal-like	
		B9_2	Basal-like	
		B12_1	Basal-like	
		B12_2	Basal-like	
L8	HR+, HER2 0	B6_1	Luminal A	Polytypic
		B6_2	Luminal B	
		B7	Luminal B	
		B10	ND	
		B13	ND	
L31	HR+, HER2 0	B6	Luminal A	Polytypic
		B9_1	Luminal B	
		B9_2	Luminal B	
		B12	Luminal B	
L33	HR+, HER2 0	B8	Luminal A	Polytypic
		B14_D	Luminal B	
		B14_I	Luminal B	
L42	HR-, HER3+	B10	Luminal A	Polytypic

		B12	HER2-Enriched	
		B13	HER2-Enriched	
L45	HR+, HER2 2+ (Not amplifie	B5_1	Luminal A	Polytypic
		B5_2	Luminal A	
		B6_D	Luminal B	
		B6_I1	Luminal A	
		B6_I2	Luminal A	
		B7_1	Luminal B	
		B7_2	Luminal B	
L48	HR+, HER2 1+	B10	Luminal B	Polytypic
		B15_1	Luminal B	
		B15_2	Luminal A	
		B16	Luminal B	
L54	HR+, HER2 0	B7_D	Luminal A	Polytypic
		B7_I	Luminal A	
		B8	Luminal A	
		B12	Luminal A	
		B12_D	ND	
		B13_2	Luminal B	
		B13_3	Luminal B	
L55	HR+, HER2 2+	B6	Luminal A	Polytypic
		B9_1	Luminal A	
		B9_2	Luminal B	
		B10	Luminal A	
L71	HR+, HER2 1+	B12	Luminal B	Polytypic
		B13	Luminal A	
		B14	Luminal B	
L73	HR+, HER2 0	B10_A1	Luminal B	Polytypic
		B10_A2	Luminal B	
		B12_1	Luminal B	
		B12_2	Luminal B	
		B14_A1	Luminal A	
		B14_A2	Luminal B	
L77	HR+, HER2 2+ (Not amplifie	B7	Luminal A	Polytypic
		B9	Luminal B	
		B10	Luminal B	
L81	TNBC	B9	Luminal A	Polytypic
		B11	Basal-like	
		B14	Basal-like	
L89	TNBC	B7	HER2-Enriched	Polytypic
		B8	HER2-Enriched	
		B10	Basal-like	

Table S.II List of all lumpectomy cases, IHC-defined subtype classification, cores/regions and the molecular subtype of each core, and the molecular subtype classification of each lumpectomy case.

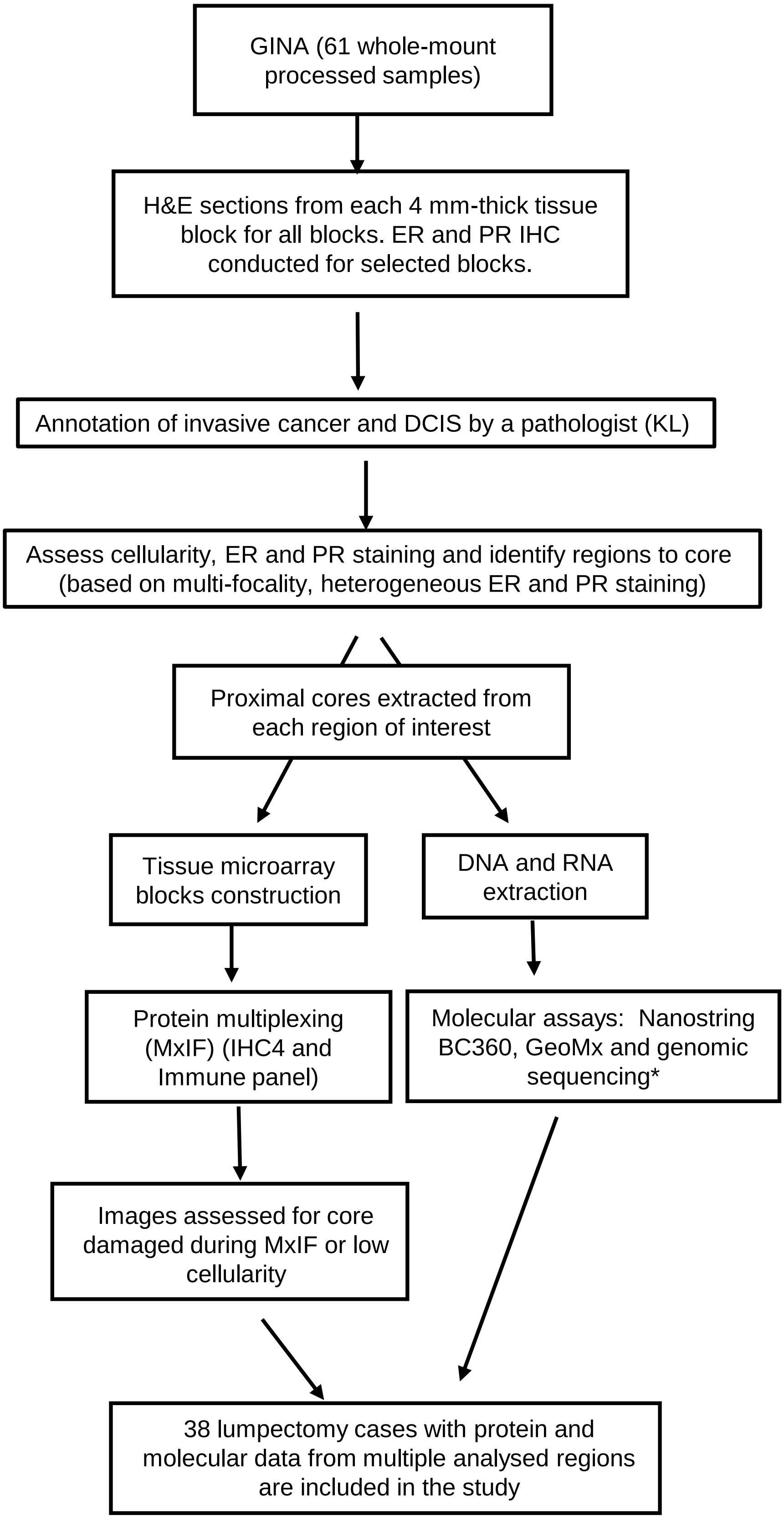

Figure S. 1 Schematic diagram showing the workflow of the study. * Only data from BC360 is included in this report.

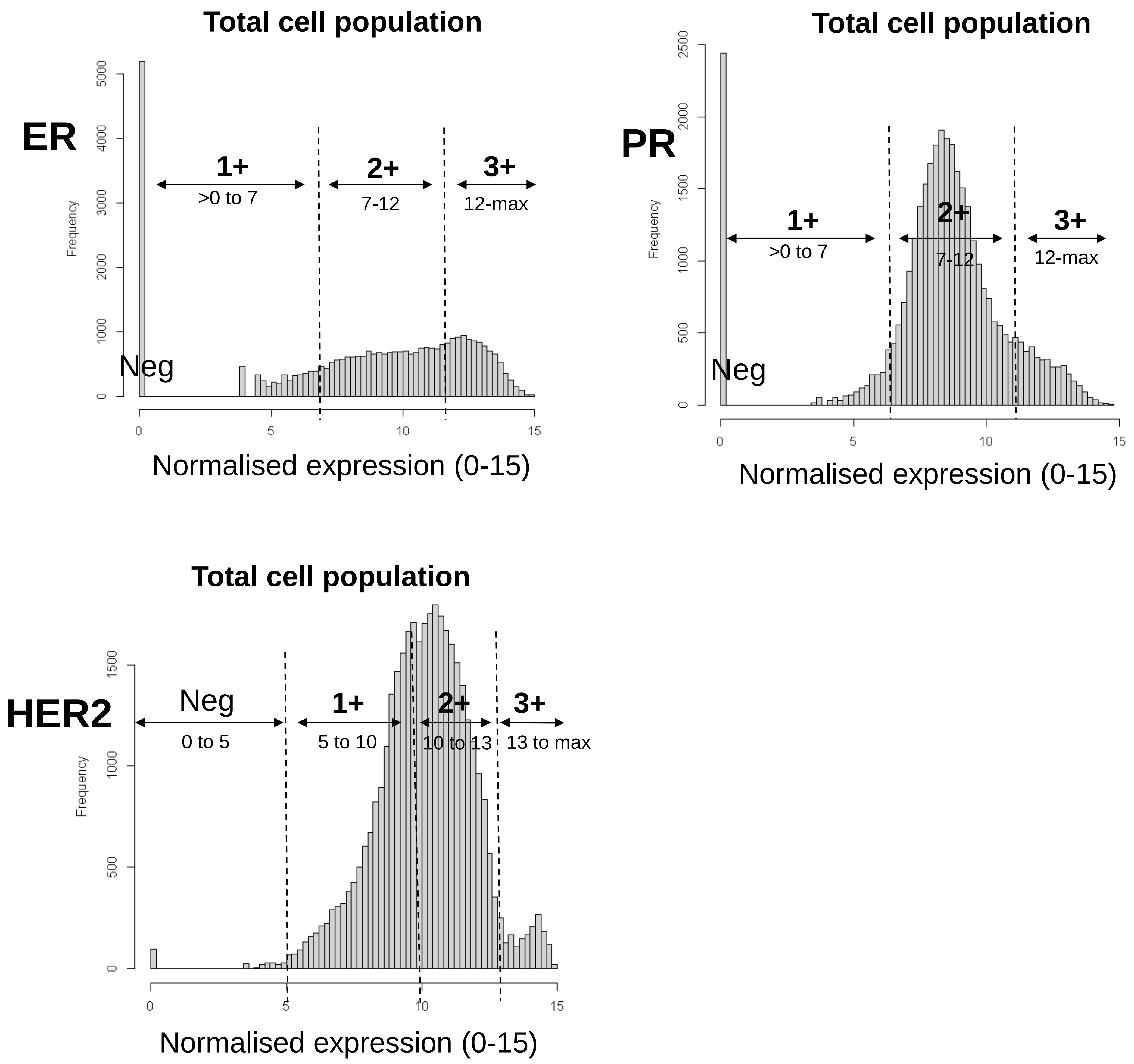

Figure S.2 Selection of cut-points of normalised expression of ER, PR and HER2 for 0 (Neg) 1+, 2+ and 3+ categories. Raw intensity expression of each protein marker per cell was log_2 transformed and normalised to a range of 0-15. Histograms showed the distribution of cells and the normalised level of protein marker in each cell, for the total cell population. For ER and PR, cells with 0 expression were classified as negative cells. Cells with normalised expression >0 to 7 are classified as 1+, 7-12 as 2+ and >12 as 3+. For HER2, cells with normalised expression 0-5 are classified as negative, >5 to 10 are classified as 1+, >10 to 13 are classified as 2+, and >13 are classified as 3+.

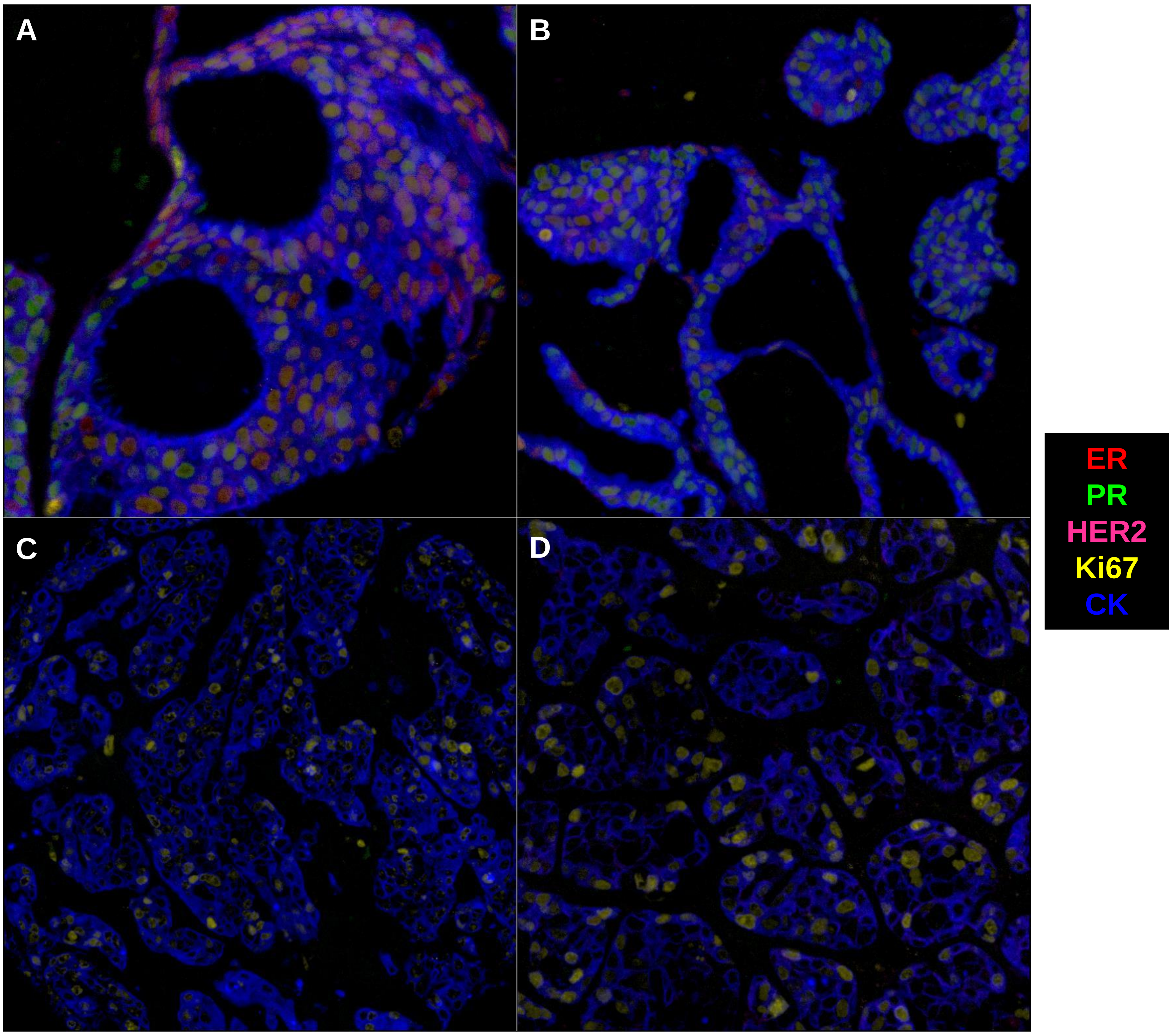

Figure S.3 Representative MxIF images from intra-tumoral images of a breast cancer molecularly subtyped as Luminal A. The degree of ER expression varied among the regions analysed, with cores A and B showed high levels of ER and PR, while cores C and D showed very little ER or PR expressions. This case showed marked levels of intra-tumoral heterogeneity, while core A is a representative example of intra-core heterogeneity with the range of ER, PR and Ki67 staining present in a single region.

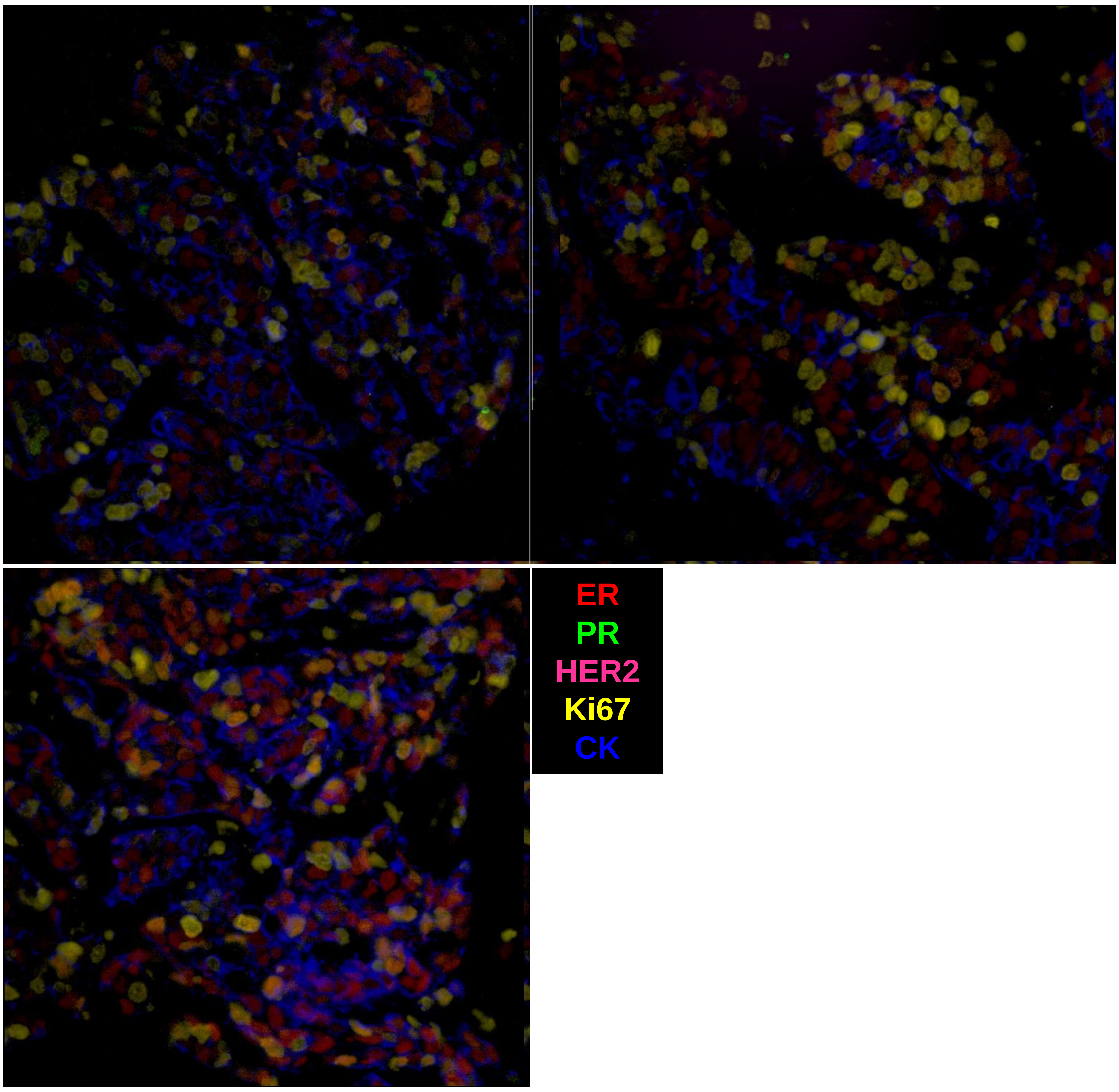

Figure S.4 Representative MxIF images from intra-tumoral images of a breast cancer molecularly subtyped as Luminal B. Two of the analysed regions (upper panels) showed lower ER expressions compared to a third region (bottom panel). The % of Ki67+ cells appeared to be similar.

A

B

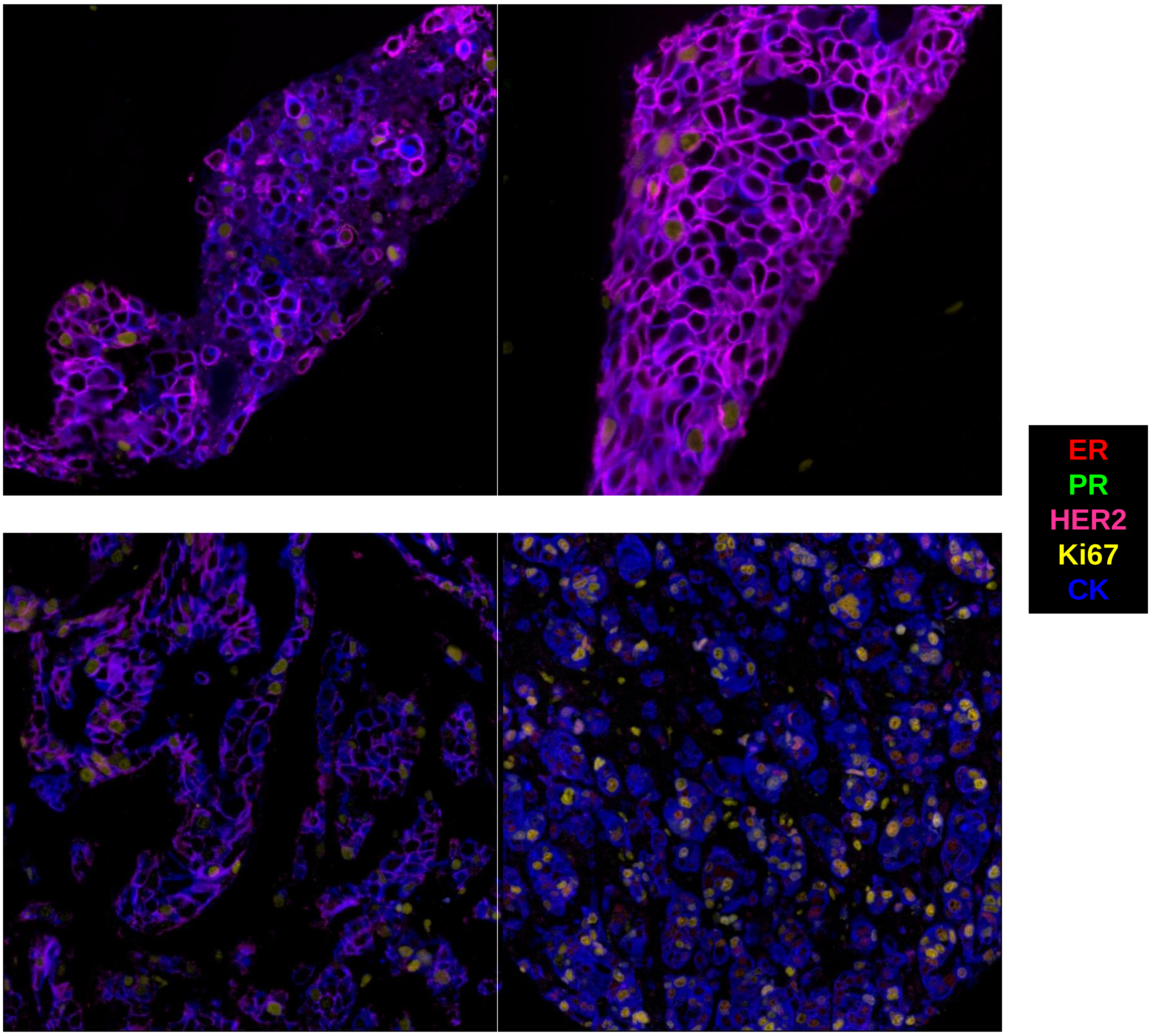

Figure S.5 Representative MxIF images from intra-tumoral images of two (IHC-scored) HER2 3+ breast cancers. (A) L42 is a molecular-polytypic cancer with cores molecularly subtyped as Luminal A and HER2-enriched. (B) L47, with all cores molecularly subtyped as HER2-enriched.

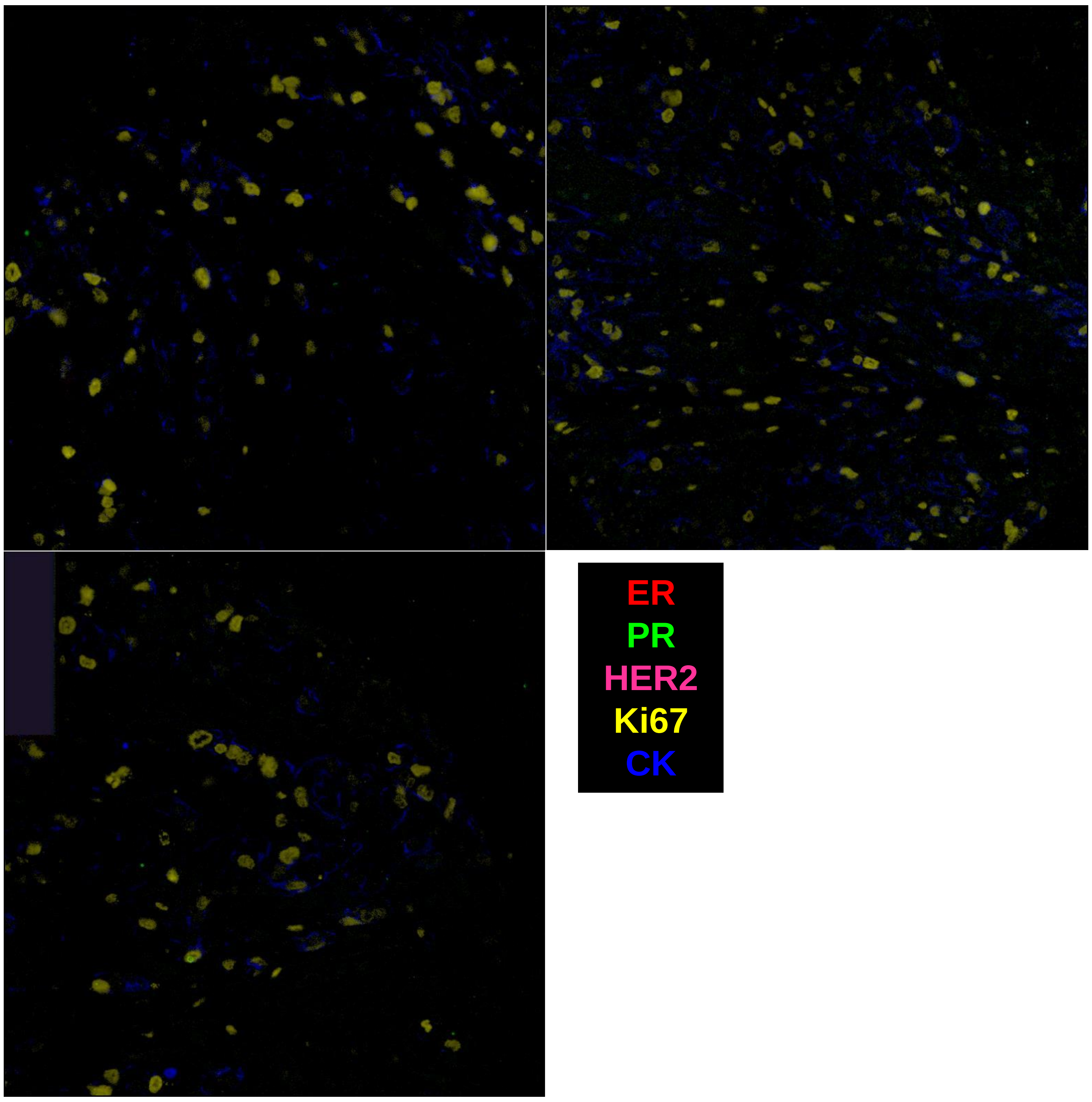

Figure S.6 Representative MxIF images from intra-tumoral images of a basal-like breast cancer.

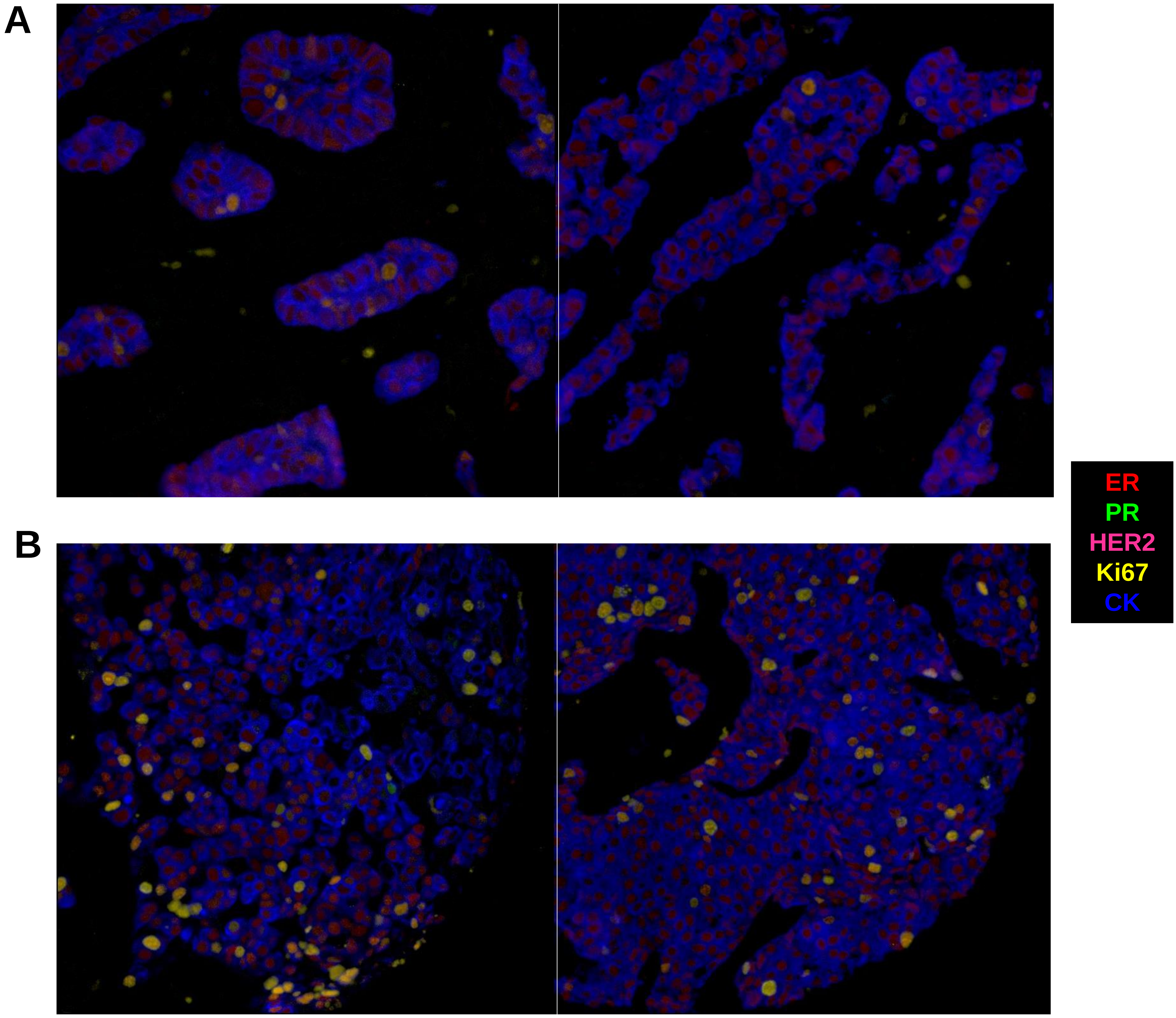

Figure S.7 Representative MxIF images from intra-tumoral images of two poly-typic breast cancers. (A) L55 has one region subtyped as Luminal B (left) and one region subtyped as Luminal A (right). (B) L73 has multiple regions subtyped as Luminal B, and one region subtyped as Luminal A.

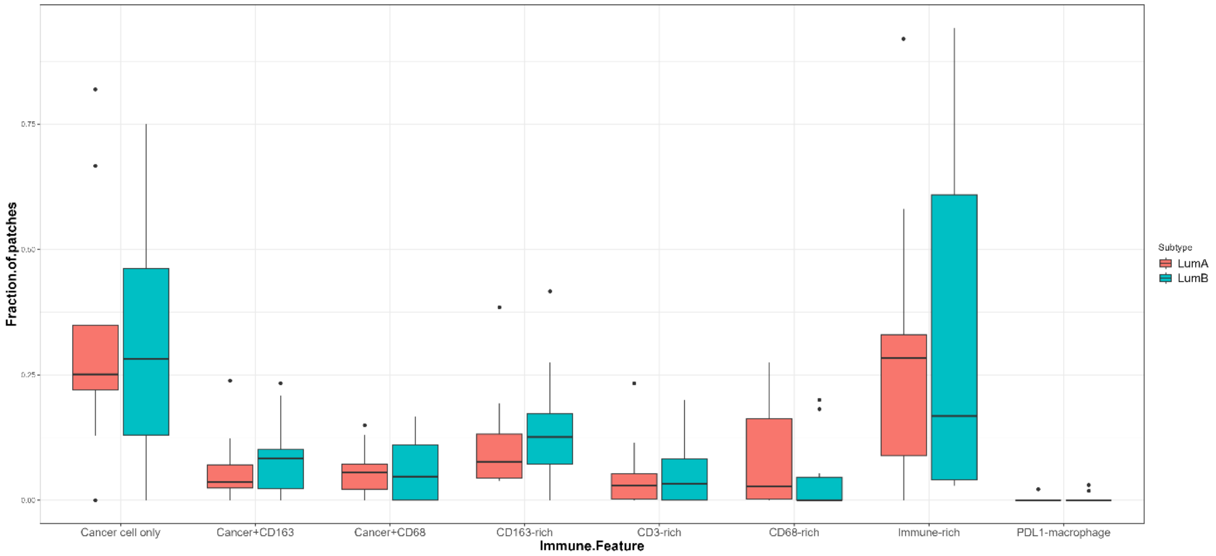

Figure S.8. Comparison of immune phenotype between LumA vs LumB cores in polytypic cancers. The fraction of image patches that exhibited the indicated spatial feature in each tissue core were presented in boxplot. N=10 LumA cores and N=12 LumB cores in polytypic cancers. Analysis with the Wilcoxon rank sum test did not show any significant difference between LumA and LumB cores.

References:

Bartley AN, Washington MK, Colasacco C, Ventura CB, Ismaila N, Benson AB 3rd, Carrato A, Gulley ML, Jain D, Kakar S, Mackay HJ, Streutker C, Tang L, Troxell M, Ajani JA. HER2 Testing and Clinical Decision Making in Gastroesophageal Adenocarcinoma: Guideline From the College of American Pathologists, American Society for Clinical Pathology, and the American Society of Clinical Oncology. J Clin Oncol. 2017 Feb;35(4):446-464. doi: 10.1200/JCO.2016.69.4836. Epub 2016 Nov 14. PMID: 28129524.

Clarke GM, Eidt S, Sun L, Mawdsley G, Zubovits JT, Yaffe MJ. Whole-specimen histopathology: a method to produce whole-mount breast serial sections for 3-D digital histopathology imaging. Histopathology. 2007 Jan;50(2):232-42. doi: 10.1111/j.1365-2559.2006.02561.x. PMID: 17222252.

Jost L. Partitioning diversity into independent alpha and beta components. Ecology. 2007 Oct;88(10):2427-39. doi: 10.1890/06-1736.1. Erratum in: Ecology. 2009 Dec;90(12):3593. PMID: 18027744.

Levine JH, Simonds EF, Bendall SC, Davis KL, Amir el-AD, Tadmor MD, Litvin O, Fienberg HG, Jager A, Zunder ER, Finck R, Gedman AL, Radtke I, Downing JR, Pe'er D, Nolan GP. Data-Driven Phenotypic Dissection of AML Reveals Progenitor-like Cells that Correlate with Prognosis. Cell. 2015 Jul 2;162(1):184-97. doi: 10.1016/j.cell.2015.05.047. Epub 2015 Jun 18. PMID: 26095251; PMCID: PMC4508757.

Rao CP, Diversity and dissimilarity coefficients: A unified approach,Theoretical Population Biology,Volume 21, Issue 1, Pages 24-43,1982.

Wang D, Cheung A, Mawdsley GE, Liu K, Yerofeyeva Y, Thu KL, Yoon JY, Yaffe MJ. A Modified Bleaching Method for Multiplex Immunofluorescence Staining of FFPE Tissue Sections. Appl Immunohistochem Mol Morphol. 2024 Nov-Dec 01;32(10):447-452. doi: 10.1097/PAI.0000000000001228. Epub 2024 Oct 7. PMID: 39370592.

Wolff AC, Hammond MEH, Allison KH, Harvey BE, Mangu PB, Bartlett JMS, Bilous M, Ellis IO, Fitzgibbons P, Hanna W, Jenkins RB, Press MF, Spears PA, Vance GH, Viale G, McShane LM, Dowsett M. Human Epidermal Growth Factor Receptor 2 Testing in Breast Cancer: American Society of Clinical Oncology/College of American Pathologists Clinical Practice Guideline Focused Update. J Clin Oncol. 2018 Jul 10;36(20):2105-2122. doi: 10.1200/JCO.2018.77.8738. Epub 2018 May 30. PMID: 29846122.
